# An Overview of the Biological Evaluation of Selected Nitrogen-Containing Heterocycle Medicinal Chemistry Compounds

**DOI:** 10.3390/ijms23158117

**Published:** 2022-07-23

**Authors:** Oluwakemi Ebenezer, Maryam Amra. Jordaan, Gea Carena, Tommaso Bono, Michael Shapi, Jack A. Tuszynski

**Affiliations:** 1 Department of Chemistry, Faculty of Natural Science, Mangosuthu University of Technology, Umlazi 4031, KZN, South Africa; ebenezer.oluwakemi@mut.ac.za (O.E.); jordaan.maryam@mut.ac.za (M.A.J.); mshapi@mut.ac.za (M.S.); 2 Department of Physics, University of Alberta, Edmonton, AB T6G 2E1, Canada; carena@ualberta.ca (G.C.); bono@ualberta.ca (T.B.); 3 Department of Mechanical and Aerospace Engineering, (DIMEAS), Politecnico di Torino, 10129 Turin, Italy; 4 Department of Oncology, Cross Cancer Institute, University of Alberta, Edmonton, AB T6G 1Z2, Canada

**Keywords:** heterocyclic compounds, nitrogen containing compounds, biological evaluations, five-membered rings, six-membered rings

## Abstract

Heterocyclic compounds are a class of compounds of natural origin with favorable properties and hence have major pharmaceutical significance. They have an exceptional adroitness favoring their use as diverse smart biomimetics, in addition to possessing an active pharmacophore in a complex structure. This has made them an indispensable motif in the drug discovery field. Heterocyclic compounds are usually classified according to the ring size, type, and the number of heteroatoms present in the ring. Among different heterocyclic ring systems, nitrogen heterocyclic compounds are more abundant in nature. They also have considerable pharmacological significance. This review highlights recent pioneering studies in the biological assessment of nitrogen-containing compounds, namely: triazoles, tetrazoles, imidazole/benzimidazoles, pyrimidines, and quinolines. It explores publications between April 2020 and February 2022 and will benefit researchers in medicinal chemistry and pharmacology. The present work is organized based on the size of the heterocyclic ring.

## 1. Introduction

Heterocyclic compounds are compounds with a cyclic ring bearing carbon and other elements, such as oxygen, nitrogen, and sulfur. The simplest of the five-membered heterocyclic compounds are pyrrole, furan, and thiophene, and the compounds contain a single heteroatom. Notably, heterocyclic compounds are the preferred class of compounds of natural origin with pharmaceutical significance. They have an exceptional adroitness to be used as diverse smart biomimetics and an active core in a complex structure, which has made them an indispensable motif in the pharmaceutical field. Several heterocyclic derivatized compounds are known. These numbers continue to expand rapidly, as heterocyclic substitutions can alter the degree of ionization of compounds in the physiological pH, resulting in changes in their basicity and lipophilicity, leading to substantial differences in pharmacokinetic properties [1,2]. For instance, heterocyclic substitutions with phenyl, anilic, or isosteric structures demonstrated improved pharmacological efficacy, pharmacokinetic properties, and lower toxicity [1,3,4]. Among different kinds of heterocyclic ring systems, nitrogen heterocyclic compounds are more abundant in nature and have considerable therapeutic importance.

**Figure 1:**
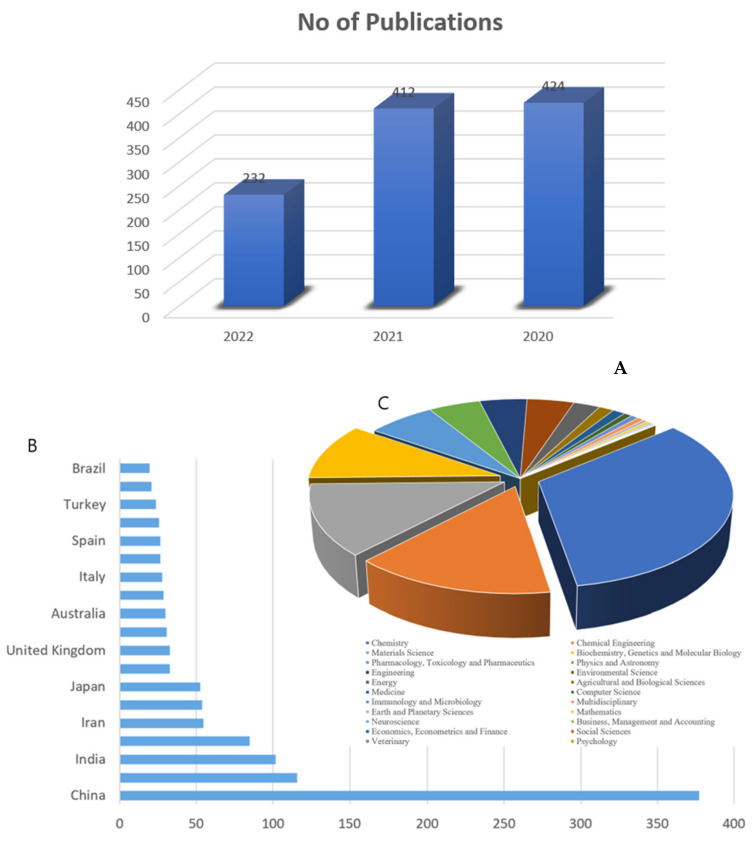
(**A**) Number of publications containing the keyword of nitrogen-containing compounds in the title of the articles drawn to the year of publication (July 1068 articles). (**B**) The country/territorial documents of most publications contain at least nitrogen-containing compounds in the title of the articles. (**C**) Documents by subject area.

The database analysis of U.S FDA-approved drugs performed by Vitaku and coworkers showed that 59% of the reported small molecule drugs contain nitrogen heterocycles [5]. Among the various nitrogen-containing heterocycles, triazoles, tetrazoles, imidazoles/benzimidazoles, pyrimidines, and quinolines have gained special attention as a broad class of abundant organic compounds with a remarkable history of therapeutic applications. These vital nitrogen heterocyclic compounds have demonstrated interesting pharmacological activity mainly as anti-tumor and kinase inhibitors [6], as well as anti-inflammatory [7,8], antimicrobial [9], antioxidant [10], antidiabetics [11], anti-convulsant [12], and dual PPAR alpha/gamma agonists [13], among many other activities. As expected, the number of publications on new medicines and biologically active compounds containing nitrogen atoms does not drastically decrement over the last two years (see Figure 1).

**Figure 2:**
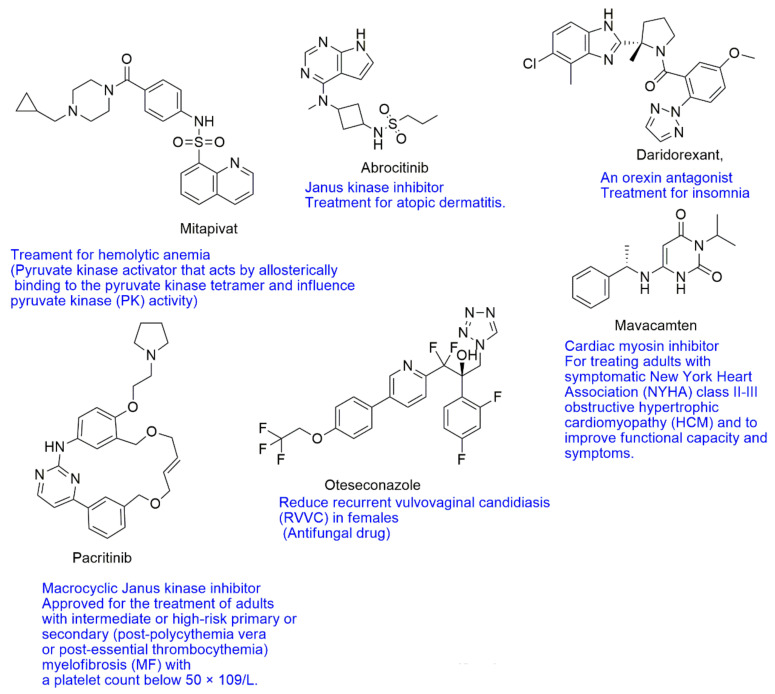
New FDA-approved nitrogen-containing heterocycle compounds.

Recently, the FDA has approved new nitrogen-containing heterocycle compounds, namely mitapivat, abrocitinib, mavacamten, pacritinib, oteseconazole and daridorexant, for therapeutic uses. Mitapivat has been demonstrated to be effective toward hemolytic anemia in adults with pyruvate kinase (PK) deficiency [14]. Pfizer has developed abrocitinib, a selective inhibitor of Janus kinase 1 (JK1), used to treat atopic dermatitis, and daridorexant, which is used to treat adult patients suffering from insomnia, characterized by problems with sleep onset and/or maintenance of sleep [14]. Daridorexant binds to and impedes the orexin receptors OX1R and OX2R (Ki = 0.47 and 0.93 nM, respectively) (see Figure 2).

**Figure 3:**
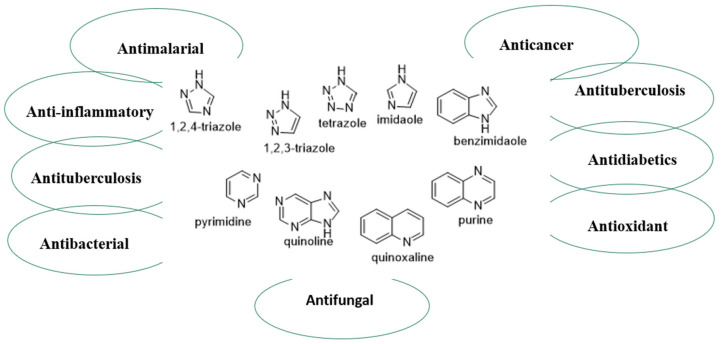
Biological assessment of nitrogen-containing compounds such as triazoles, tetrazole, imidazole, benzimidazole, pyrimidine, quinoline, quinoxaline, and purine.

This review highlights important pioneering studies in the biological assessment of nitrogen-containing compounds such as triazoles, tetrazoles, imidazoles/benzimidazoles, pyrimidines and quinolines. It covers the short but dynamic period of time between 2020 and 2022 where much of the development in this area has taken place. It is our hope that this work will be of benefit to researchers in the fields of medical chemistry and pharmacology (see Figure 3). This present work is organized based on the size of the heterocyclic ring as described below:

- Five-membered heterocyclic compounds
  - Triazole (1,2,3-triazole and 1,2,4-triazole)
  - Tetrazole
  - Imiazole/Benimidazole
- Six-membered heterocyclic compounds
  - Pyrimidine
  - Quinoline
  - Quinoxaline
  - Purine

## 2. Five-Membered Heterocyclic Compounds

### 2.1. Triazole

Heterocyclic systems continue to generate considerable interest due to their broad spectrum of biological activities. Triazole and its derivatives have increased in importance as they represent the structural characteristics of many bioactive compounds. They are known to be included in the structure of many medications, namely, itraconazole, fluconazole, voriconazole, ribavirin, mubritinib, and posaconazole, amongst others. The triazole ring features three nitrogen atoms in the five-membered aromatic ring and is significantly isomeric based on the placement of nitrogen atoms in the ring. Triazole and its derivatives can interact with various enzymes and receptors in the biological system through the diversity of non-covalent interactions, thereby presenting versatile biological activities [15]. Two significant forms of triazole include 1,2,3-triazole and 1,2,4-triazole. Extensive research on triazole and its derivatives has shown the major pharmacological importance of this heterocyclic nucleus.

### 2.2. 1,2,3-Triazole

**Figure 4:**
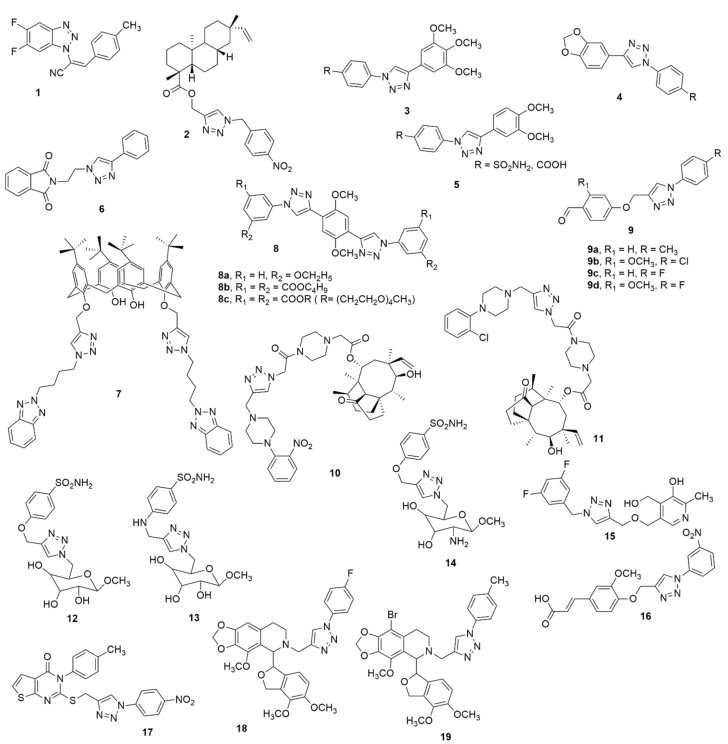
Structures of most active analogues of 1,2,3-triazole molecule.

Riu et al. [16] designed and synthesized new and improved benzotriazole–acrylonitrile derivatives incorporating two halogen atoms in positions 5′ and 6′ on the benzotriazole moiety. The compounds were further subjected to biological evaluation. Compound **1** was the most potent in the new series of derivatives. The in vitro XTT assay, flow cytometry analysis, and immunostaining performed on HeLa cancer cells treated with **1** displayed a significant antiproliferative effect, with an IC_50_ value of 3.2 µM. It was demonstrated to block the cells in G2/M-phase, and subsequently cause cell division defects. Additionally, *β*-tubulin staining validated the microtubule as being a potential molecular target of **1**, while the colchicine competition assay indicated that compound **1** vies with colchicine for the binding site on tubulin. Kasemsuk et al. [17] synthesized a novel series of acanthoic acid analogues by substituting a carboxyl functional group of acanthoic acid with methyl ester hybrids bearing a triazole ring through esterification and the CuAAC reaction and considered their cytotoxic activity against cholangiocarcinoma cell lines. Among the evaluated compounds, **2** showed the most significant activity with an IC_50_ value of 18 μM against the KKU-213 cell line, which was eight-fold more effective than acanthoic acid. An assessment of anti-inflammatory, ulcerogenic, platelet activation activities, and the molecular docking studies of COX-2 and P-selectin of the 1,4-diaryl-1,2,3-triazole hybrids has been recently published [18]. The analogs **3**–**5** exhibited anti-inflammatory activity and lacked the induction of gastric lesions in mice when compared to the reference drug, indomethacin. The reduction of polymorphonuclear cells’ influx into the peritoneal cavity caused by carrageenan indicated that these active compounds could favor a dual inhibition of COX-2 and 5-LOX enzymes. The hybrids caused a reduction in the expression of P-selectin, which may be liable to mitigate inflammation and thromboembolic events. The molecular docking study with P-selectin showed crucial interactions with the amino acid residue Tyr 48 (see Figure 4).

Holanda et al. [19] utilized the click chemistry approach to efficiently synthesize alkyl-substituted phthalimide 1*H*-1,2,3-triazole derivatives and evaluated their leishmanicidal potential against *Leishmania amazonensis* and *Leishmania braziliensis*. Compound **6** selectively inhibited the increase and existence of promastigote and amastigote forms of *L. amazonensis* and *L. braziliensis*. The molecular docking study indicates that compound **6** is a potential inhibitor of parasite sterol 14α-demethylase due to its interaction with the heme groups of this enzyme. The amalgamation of benzotriazoles and calixarenes through click chemistry produces a new class of p-tert-butyl-calix [4] arene tethered benzotrizolyl dendrimers, which has been reported together with their biological evaluation [20]. Compound **7** was found to be the most potent antibacterial and anti-biofilm agent against drug-resistant and slime-generating organisms with no detected cytotoxicity to the mammalian cell line. Melah et al. [21] reported the design, synthesis, and in vitro antiproliferative activities of novel mono- and bis-1,2,3-triazole molecular hybrids. Compound **8a** exhibited potent activity against HepG-2 when compared to the standard reference anticancer drug doxorubicin whereas compounds **8b** and **8c** demonstrated significant toxicity on the RPE-1 human normal cells. Sahin et al. [22] reported the design and synthesis of several 1,2,3-triazole compounds with aldehyde functional groups. The synthesized compounds were further examined for their antioxidant, anti-cancer, and α-amylase enzyme activity. The DPPH radical scavenging studies showed that all the compounds have a higher activity than the standard BHT and β-carotene. Compound **9a** displayed almost the same scavenging effect with β-carotene and BHT. Compound **9b** (IC_50_ = 165 μg/mL) was almost 5.5-fold superior to acarbose regarding its α-amylase inhibition activity. The synthesized compounds were screened for anti-cancer activities against the HeLa cell line. Compound **9c** and **9d** with IC_50_s of 50.12 and 57.07 μg/mL, respectively, displayed mild antitumor activity when compared to cisplatin against the HeLa cell line (see Figure 4).

A novel series of pleuromutilin derivatives bearing piperazine and 1,2,3-1H-triazole structures were synthesized by click chemistry methodology under mild conditions [23]. The compounds were further investigated for their MIC and MBC against methicillin-resistant *S. aureus* (ATCC 43300), *S. aureus* (ATCC 29213), *S. aureus* (AD3), *S. aureus* (144), and *E. coli* (ATCC 25922). Compounds **10** and **11** displayed more significant antibacterial activity than other compounds. Compound **10** exhibited rapid kinetics of its bactericidal activity against MRSA and had a longer PAE than tiamulin. The in vivo antibacterial effectiveness of **11** was further studied in a neutropenic murine thigh infection model. The outcomes revealed that compound **10** exhibited more effective in vivo antibacterial activity than tiamulin. In addition, **10** exhibited low to moderate repressing effects on CYP1A2, CYP2E1, CYP2D6, and CYP3A4 enzymes. The design, synthesis, and biological evaluation of a panel of novel aromatic sulfonamides linked to a hydrophilic sugar-tail moiety using rigid 1,2,3-triazole as a spacer have been reported [24]. The newly designed compounds were investigated in vitro and an efficient inhibition against all three CA isoforms, especially the tumor-associated hCA IX, was observed. All the glycoconjugate sulfonamide derivatives exhibited superior inhibitory activity. Compound **12** was the most effective and selective inhibitor of hCA IX with an inhibitory constant (IC_50_) value of 7 nM, being four-fold superior to acetazolamide (AAZ) whose IC_50_ value is 30 nM. In both hypoxic and normoxic conditions, almost all the compounds exhibited moderate antiproliferative activities against two cancer cell lines (HT-29 and MDA-MB-231). Notably, **12** exhibited superior antitumor activity and cytotoxic activity. In addition, the combined therapy evaluation found noticeable decreases (20–35%) in doxorubicin IC_50_ values in MDA-MB-231 cancer cells in a hypoxic environment in the presence of compounds **12**–**14**, carbonic anhydrase inhibitors, when compared to single therapy (doxorubicin) (see Figure 4).

Multi-target natural product-pyridoxine-based derivatives were designed, synthesized, characterized, and evaluated as potential anti-Alzheimer agents [25]. Among the tested compounds, **15** acted as a potent acetylcholinesterase (AChE) inhibitor, (IC_50_ = 1.56 ± 0.02 mM) and exhibited antioxidant activity, having an ORAC-FL value of 1.21 ± 0.28, which is comparable to Trolox. The docking studies showed interactions between the peripheral anionic site of the enzyme (PAS site) with the hydrophobic amino acids Tyr 124 and Phe 338 and the triazole nucleus of **15**. Compound **16** has been reported to selectively inhibit carbonic anhydrase IX (CAIX) with an IC_50_ value of 24 nM [26]. The in silico analysis revealed the binding of **16** with the catalytically significant amino acid residues of CAIX. Additionally, cell-based studies showed that **16** prevents the activity of CAIX, reduces the epithelial-to-mesenchymal transitions, induces apoptosis, and obstructs cell migration and colonization potential of cancer cells (see Figure 4).

Suryanarayana et al. [27] reported the synthesis of thieno[2,3-*d*]-pyrimidine fused 1,2,3-triazole scaffolds and their antioxidant activity. Compound **17** exhibited good antioxidant activity against DPPH scavenging with an IC_50_ value of 8.161 μM, as compared to the standard drug ascorbic acid (IC_50_ = 3.073 μM). Notably, electron-removal groups in the *para*-position of the derivatives provided excellent scavenging capacity for all three scavenging methods when compared to the electron-donating groups. Twenty novel 1,2,3-triazole noscapine derivatives have been synthesized using noscapine as a precursor and were further evaluated for their biological activity [28]. Interestingly the combination of computational and experimental evaluation revealed two potent compounds **18** and **19**, (K_D_ = 21.5 ± 6.15 and 36.9 ± 4.24 nM, respectively) compared to noscapine (K_D_ = 579.0 ± 18.7 nM) (see Figure 4).

**Figure 5:**
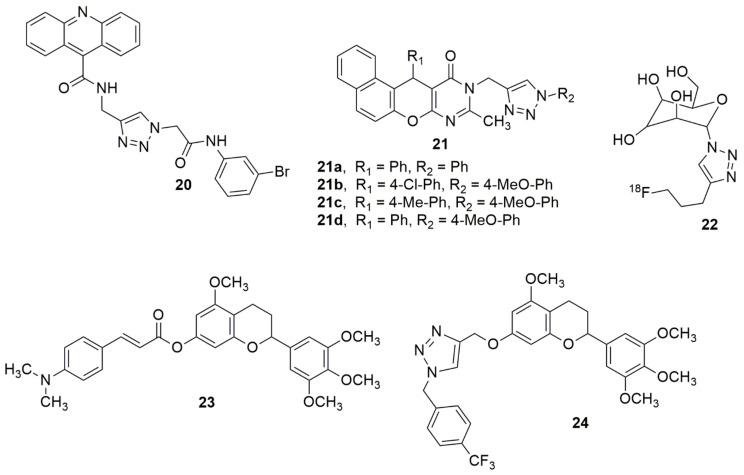
Chemical structures of 1,2,3-triazole hybrids with anti-diabetics (**20**) and anti-Alzheimer activities (**21,23,24**).

In an attempt to introduce new scaffolds as potent α-glucosidase inhibitors, Sepehri et al. [29] reported a new series of acridine-9-carboxamide-1,2,3-triazole-*N*-phenylacetamide derivatives. They were screened for their in vitro α-glucosidase inhibitory activities. Among the screened compounds, **20** exhibited a superior potency with an IC_50_ of 80.3 ± 0.9µM compared to the standard drug acarbose (IC_50_ = 750.0 ± 10.5µM). Cherif et al. [30] designed and synthesized some new hybrid compounds through the amalgamation of a pyranopyrimidinone moiety with 1,2,3-triazole pharmacophore via 1,3-dipolar cycloaddition using different arylazides. Compounds **21a**–**21d** showed strong capacities of cholinesterase inhibition with IC_50_ values of 6.7 ± 0.2, 8.4 ± 0.4, 7.8 ± 0.2 and 9.1 ± 0.1 µM, respectively. Compound **22** has been reported to be a feasible positron emission tomography (PET) probe that can offer a better understanding of the ASGPR (asialoglycoprotein receptor)-related liver disease [31] (see Figure 5).

Shi and co-workers designed and synthesized 7-*O*-modified galloyltricetiflavan hybrids containing cinnamate, benzoate, phenyl sulfonate, and 1,2,3-triazole scaffolds [32]. Meanwhile, all synthesized compounds were examined for the inhibition of AChE/BuChE (butyrylcholinesterase) and anti-A*β* aggregation activity. Among the evaluated compounds, **23** exhibited the best inhibition of A*β* aggregation (78.81% at 20 μM), and superior AChE inhibitory potencies (IC_50_, 0.56 μM). Compound **24** exhibited the highest BuChE activity (IC_50,_ 5.77 μM). Compounds **23** and **24** exhibited high potent protective capabilities than Trolox against H_2_O_2_- induced SH-SY5Y cell injuries. The observed potent compounds lacked visible toxicity in SH-SY5Y cells and could slightly increase SHSY5Y cell viabilities. Hence, **23** and **24** were reported as promising multi-functional agents for the treatment of Alzheimer’s disease (see Figure 5).

**Figure 6:**
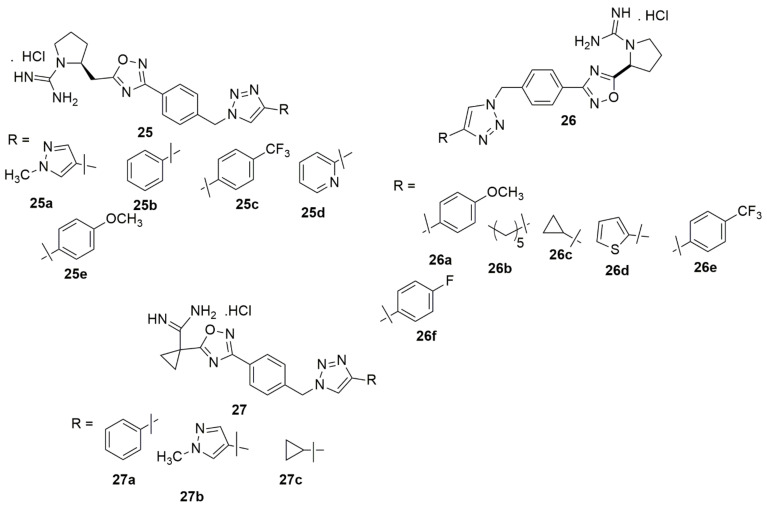
New 1,2,3-triazole series evaluated for SphK2 inhibitory activity using an ADP-Glo kinase assay.

Tangadanchu et al. [33] designed, synthesized, and evaluated a series of eighteen new 1,2,3-triazole compounds and evaluated their sphingosine kinase-2 (SphK2) inhibitory activity using an ADP-Glo kinase assay. The in vivo anti-tumor bioactivity was further explored. Many of the screened compounds exhibited potent selectivity for SphK2 over SphK1. Compounds **25a**, **26a**–**26c**, **27a**, and **27b** were potent towards SphK2 with IC_50_ values of 0.234, 0.266, 0.254, 0.248, 0.261, and 0.269 µM, respectively, whereas the compounds **25a**, **25d**, **25e**, **26b**–**26f**, **27a**–**27c** showed a high selectivity for SphK2 versus SphK1. In addition, compounds **25b**–**25c**, and **27e** exhibited superior antitumor activity for the human malignant glioblastoma tumor U-251 MG cell line when compared to ABC294640 (see Figure 6).

**Figure 7:**
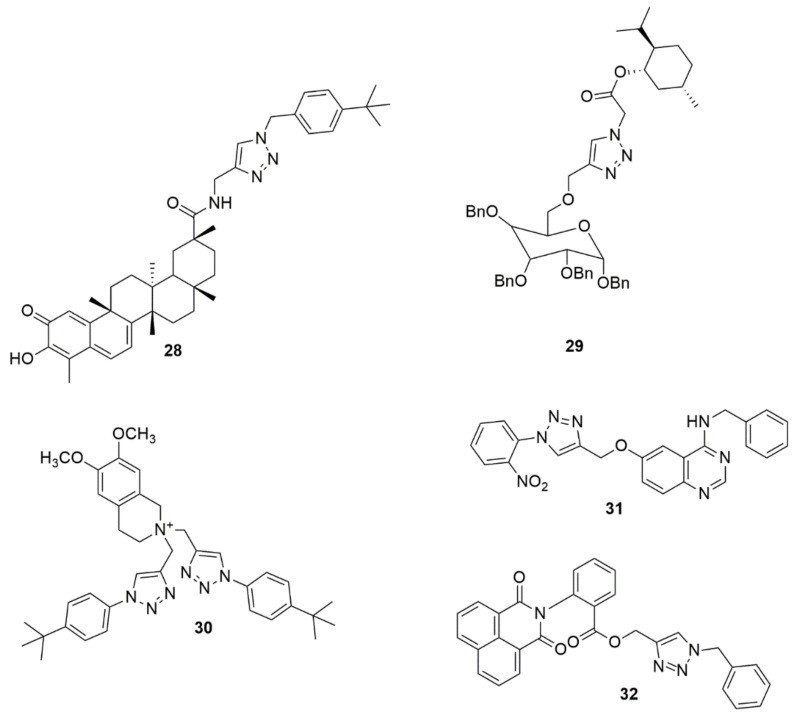
Chemical structures of 1,2,3-triazole hybrids with promising biological activities.

Compound **28** has been reported to exhibit potent antiproliferative activity in U251 cells with an IC_50_ value of 0.94 µM, and it significantly inhibited the colony formation and migration of U251 cells [34]. Chaidam et al. [35] designed and synthesized a series of novel 1,6-bis-triazole-2,3,4-tri-*O*-benzyl-*α*-D-glucoside derivatives. The synthesized compounds were screened for their anti-diabetic activity. Among the examined compounds, **29** showed superior inhibitory activity with IC_50_ values of 3.73 μM, which was 39-fold higher than that of acarbose. Notably, the presence of the ester functional group and menthol moiety played a significant role in its biological activity due to increasing polarity, which enhanced binding against *α*-glucosidase. The library of the cationic tetrahydroisoquinoline–triazole compounds has been synthesized using the copper(II)-catalyzed azide–alkyne cycloaddition [36]. The compounds were evaluated for their antibacterial activity. Compound **30** potently inhibits Gram-positive pathogens and *M. tuberculosis*. The potent compound inhibited *M. tuberculosis* H37Rv at 6 μg/mL MIC. Compound **30** resulted in lysis and a bulging/swelling phenotype, suggesting compound **30** may target cell wall or membrane homeostasis. The cell passage test demonstrated that *S.aureus* did not develop resistance against **30** even at sub-inhibitory concentrations. A new series of quinazoline–triazole hybrid compounds have been designed, synthesized and evaluated for their anti-AChE activity by Le-Nhat-Thuy and co-workers [37]. Most of the synthesized compounds showed moderate to good AChEI activity. Among the evaluated compounds, *N*-benzyl-6-((1-(2-nitrophenyl)-1*H*-1,2,3-triazol-4-yl)methoxy)quinazolin-4-amine **31** was shown to have the highest inhibitory activity with an IC_50_ value of 0.23 μM (see Figure 7).

Bengam et al. [38] designed and synthesized novel naphthalimide-1,2,3-triazole tethered heterocycles and evaluated their in vitro anti-inflammatory properties. Among the compounds tested, **32** displayed inhibitions comparable to the reference compound (diclofenac sodium). The compound **32** showed 95.25% inhibition, and the reference drug showed 97.89% inhibition at 200 μM. The molecular docking analysis showed that the triazole ring **32** hydrogens bonded with the amine group of TYR385 and the amine group of TRP387 with the carbonyl group of anthranilic moiety (see Figure 7).

**Figure 8:**
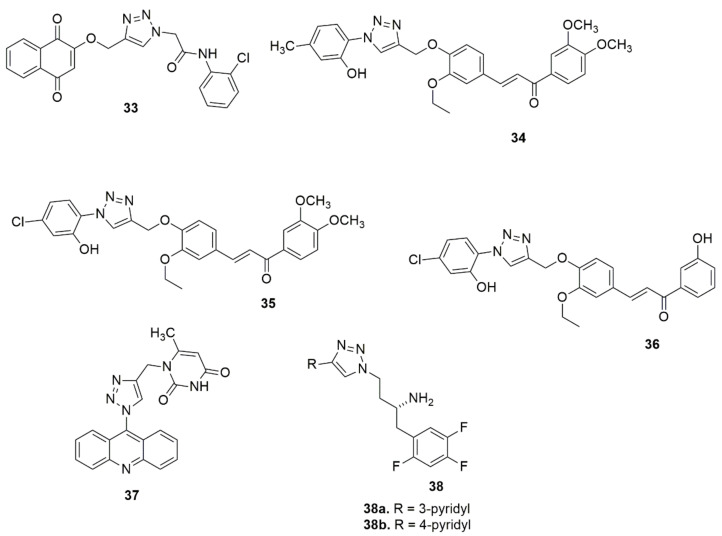
Chemical structures of 1,2,3-triazole hybrids with biological activities.

Hosseini et al. [39] used a molecular hybridization strategy to design a novel series of naphthoquinone derivatives bearing an acetamide–triazole moiety as novel AChE and BuChE inhibitors. Among the synthesized compounds evaluated for biological activity, **33** with an *ortho*-chlorine substituent exhibited the most potent AChE and BuChE activity with K_i_ values of 10.16 and 8.04 nM, respectively, compared to the standard compound Tacrine (K_i_ = 70.61 and 64.18 nM). Notably, the insertion of a chlorine atom at the *ortho* position enhanced the ChEs’ inhibition. Compound **33** was well fitted in the AChE and BuChE binding pocket via strong hydrogen bond interactions with the significant residue of each enzyme. Synthesis of a series of novel 1,2,3-triazole tethered chalcone derivatives and their cytotoxic activity against the human breast cancer cell line (MCF-7), cervical cancer (HeLa), and MDA-MB-231 cell lines have been reported [40]. In vitro cytotoxic activity evaluated using an MTT assay showed that all the synthesized compounds exhibited moderate to substantial cytotoxic activity. Compounds **34**, **35**, and **36** exhibited potent cytotoxic activity with IC_50_ values lower and comparable to cisplatin. Compounds **35** showed the best cytotoxic activity on MCF-7, with IC_50_ values of 1.27 and 0.02 µM at 24 and 48 h, respectively. Compounds with a chloro and methoxy substituent at different positions displayed promising activity. Abdel-Hafez and co-workers [41] designed, synthesized and hybridized acridine and coumarin derivatives, and evaluated their in vitro cancer cell growth inhibition activity. Among the evaluated compounds, **37** presented a good anticancer profile against MCF7 and DU-145 with IC_50_, values of 2.7 and 26.1 µM, respectively, comparable to doxorubicin (IC_50_ values = 2.0 and 14.2 µM). Compound **37** displayed greater inhibitory activity against topoisomerase (IIB) (IC_50,_ 0.52 µM) when compared with doxorubicin (IC_50_ = 0.83 µM). The novel compounds **38a** and **38b** have been reported as DPP-4 inhibitors with IC_50_ values of 28 and 14 nM, respectively [42] (see Figure 8).

### 2.3. 1,2,4-Triazole

**Figure 9:**
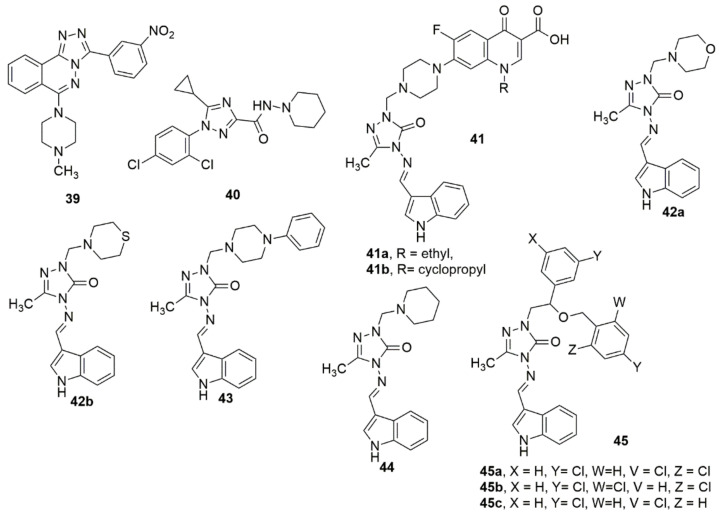
Chemical structures of analogues of 1,2,4-triazole molecule with promising biological activities.

A novel series of 3-aryl-6-(*N*-methylpiperazin)-1,2,4-triazolo [3,4-*a*]phthalazines have been synthesized through a facile and economical one-pot copper-catalyzed method from 4-chloro-1-phthalazinyl-arylhydrazones as potential anticancer agents [43]. Most of the evaluated compounds showed anticancer activity against PC-3, MCF-7, and SKBr3 cancer cell lines. Interestingly, **39** displayed apparent anticancer activity with reduced toxicities, and appropriate selectivity indexes, and acted as potassium channel blockers. Meanwhile, the fused triazolo-phthalazine hybrid and the NO_2_ substituent enhanced the biological activity. Compound **40** was reported to be the most effective inhibitor of CB1 activity (0.644 µM) and showed the most effective selectivity of CB2/CB1 (>311) [44]. However, the lack of penetration of **40** through the blood–brain barrier similar to Rimonabant in the MDCK-mdr1 permeability analysis can result in a secondary effect on the CNS. This is apparently caused by the small, obstructed, hydrophobic cyclopropyl group of 1,2,4-triazole. A library of new indole-3-carbaldehyde-triazole hybrids has been synthesized under conventional and microwave-mediated conditions [45]. The antimicrobial assessment of the compounds showed that **41a**–**41b**, bearing a fluoroquinolone scaffold and **42**–**44**, displayed remarkable activities on Gram-positive and Gram-negative bacteria with MIC values < 0.24 μg/mL. In addition, compounds **41a**–**41b**, **42**–**44**, and **45a**–**45c** showed excellent antifungal activities (see Figure 9).

**Figure 10:**
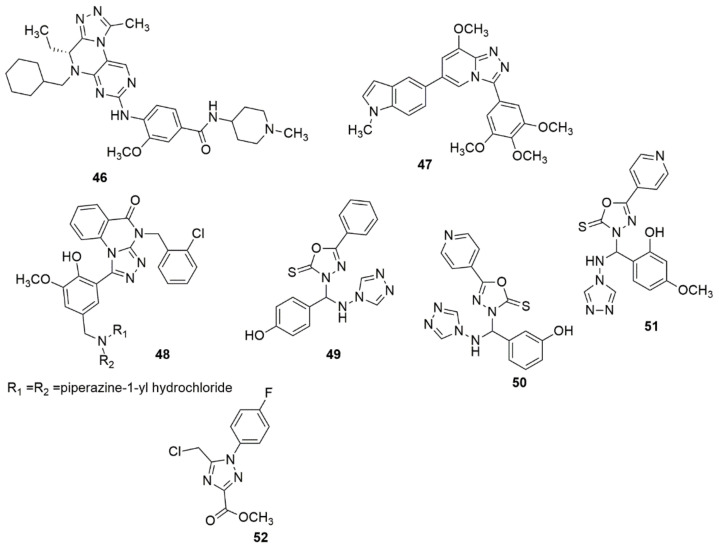
Chemical structures of 1,2,4-triazole hybrids with promising biological activities.

Wang et al. [46] reported a series of interesting compounds and their biological activity evaluation with respect to this tricyclic chemotype of dual PLK1/BRD4 inhibitors to corroborate their effectiveness as anticancer agents. The compounds were synthesized based on the core structure of BI-2536 (PLK1 inhibitor). Among the evaluated compounds, **46** displayed excellent activity for PLK1 (IC_50_ = 22 nM) and BRD4 (IC_50_ = 109 nM), with promising antiproliferative activity against a panel of cancer cell lines. Meanwhile, compound **46** displayed equipotent activity with PLK1 (IC_50_ = 22 nM) and BRD4 (IC_50_ = 109 nM). The SARS detailed that a bulkier group on the piperazine ring will enhance the stabilized potency between PLK1 and BRD4. Depending on the concentration, the potent compound greatly increased the number of Annexin V/PI-positive MV4-11. This indicates its apoptotic induction effect in cancerous cells, which has also been confirmed by the ascending regulation of apoptosis-associated proteins, including cleaved caspase-3 and cleaved PARP, along with the regulation of the anti-apoptosis protein Bcl-2. In addition, **46** demonstrated favorable in vivo anti-tumor activity with 66% tumor growth inhibition (TGI) at a 60 mg/kg dose without evident toxicity. Wu and coworkers [47] synthesized indole-based [1,2,4]triazolo[4,3-a]pyridine hybrids and screened them for their antiproliferative activities, tubulin polymerization inhibition, and cell cycle arrest/apoptosis-initiating effects. In particular, four cancer cell lines, including human cervical cancer cells (HeLa), human adenocarcinoma epithelial cells (A549), human breast cancer cells (MCF-7), and human colon cancer cells (HCT116), were employed in the standard 3-(4,5-dimethylthiazol-2-yl)-2,5-diphenyltetrazolium bromide (MTT) assay. Compound **47** bearing an *N*-methyl-5-indolyl substituent at the C-6 position of the [1,2,4]triazolo[4,3-a]pyridine moiety displayed superior activity against all the tested cell lines. In addition, compound **47** displayed potent inhibitory activity with respect to tubulin polymerization with an IC_50_ value of 1.64 ± 0.11 µM, comparable to CA-4 with an IC_50_ of 1.24 ± 0.08 µM. The primary mechanism of action (MOA) studies demonstrated that **47** could inhibit the proliferative of cancer cells by inducing cell cycle arrest at the G2/M phase and cellular apoptosis in HeLa cells in a dose-dependent manner. The active compound was also detected to have the potential ability to inhibit tumor cell migration and metastasis (see Figure 10).

A series of novel triazoloquinazolinone derivatives were designed, synthesized, and evaluated for their inhibitory activity toward the SHP2 protein enzyme [48]. Among the evaluated compounds, **48** displayed the highest inhibitory activity against the SHP2 protein at 10 µM (31.84% inhibition) compared with SHP244. **48** exhibited superior antitumor activities, with an IC_50_ value of 14.67 µM against A375 cells. The SARs revealed that derivatives with hydroxyl substituents at the two-position of the phenyl ring showed significantly higher activity than derivatives with substituents at the four-positions. In addition, the insertion of electron-withdrawing groups, namely methoxy groups, exhibited enhanced inhibitory activity. Compounds **49**–**51** have been reported by Jain et al. as being promising for the management of cognitive dysfunction [49]. Li et al. [50] designed and synthesized 1,2,4-triazole-3-carboxylates derivatives; the obtained products were subjected to in vitro NO production and cyclooxygenase COX-1/COX-2 inhibition assays. Notably, compound **52** showed the significant inhibition of NO, COX-2 (IC_50_ of 2.87 and 17.9 nM), and substantial selectivity (COX-1/COX-2 = 1080). Meanwhile, compound **52** (5 mg/kg) displayed significant in vivo anti-inflammation and gastric protection results, including paw edema, chemokines, and histological experiments, compared to Indomethacin (10 mg/kg). The presence of a fluorine atom enhanced the COX-2 inhibitory activity. The docked complex of **52** showed a comparable interaction landscape with celecoxib in the active COX-2. The active compound formed three hydrogen bond interactions with His75, Leu338, and Phe504 and eight van der Waals interactions with Val335, Leu338, Ser339, Try341, Phe504, Val509, Gly512, and Ala513, respectively (see Figure 10).

**Figure 11:**
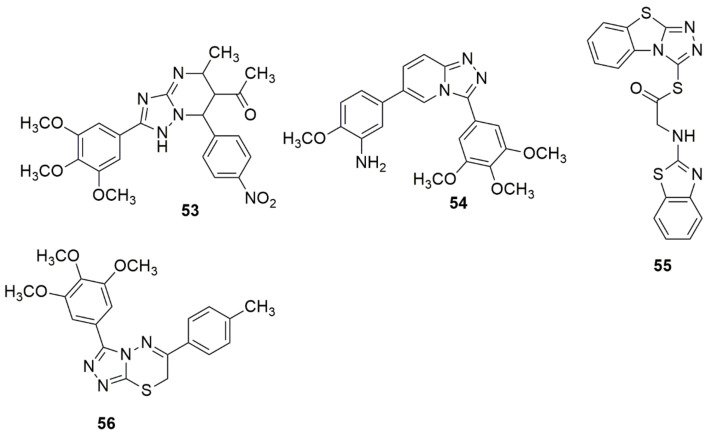
Chemical structures of 1,2,4-triazole hybrids with significant anticancer activities.

A series of novel 3,4,5-trimethoxyphenyl substituted [1,2,4]triazolo[4,3-a]pyridines were designed and synthesized on the basis of triazolopyrimidine **53** as the core compound [51]. The in vitro antiproliferative efficacy of synthesized novel 1,2,4-triazolo[4,3-a]pyridine derivatives were screened against different cancer cell lines using the (MTT) assay. Compound **54** bearing 3-amino-4-methoxyphenyl moiety exhibited the highest activity with an IC_50_ value of 12 nM, equipotent with CA-4(12nM). Also, **54** was 62-fold superior to compound **53**. The active compound **54** also exhibited potent activities against A549, MCF-7, and T47D. The MOA analysis result showed that **54** significantly blocked the cell cycle at the G2/M phase, induced apoptosis in a dose-dependent manner, and disrupted microtubule networks. Compound **54** also exhibited better anti-tubulin activity than CA-4 (see Figure 11).

Compound **55** has been reported to have substantial apoptosis-inducing activity in A549 cells and inhibited the activity of CDK2/Cyclin A1 with an IC_50_ value of 4.65 µM [52]. Ma et al. [53] synthesized a novel series of triazolothiadiazine hybrids via the ring-merging approach. The compounds were examined for their in vitro antiproliferative efficacy toward a human colon cancer cell line (HT-29) using an MTT assay. Among the evaluated compounds, **56** demonstrated excellent selectivity over the normal human embryonic kidney HEK-293 cells (IC_50_ > 100 µM). Compound **56** strongly blocked tubulin polymerization and disrupted intracellular microtubule networks. Compound **56** effectively inhibited the tumor growth of an A549 lung cancer xenograft mouse model without evident signs of toxicity in the in vivo experimentation (see Figure 11).

### 2.4. Tetrazole

**Figure 12:**
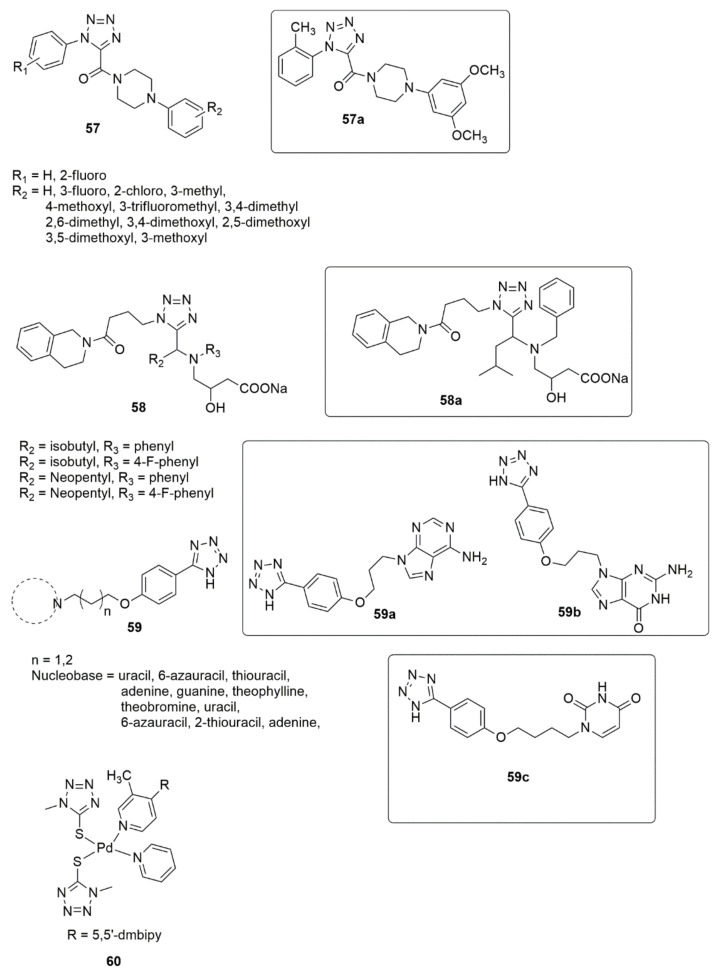
Chemical structures of active tetrazole hybrids with promising anticancer activities.

Interest in tetrazole derivatives has increased considerably over the past few decades because of the virtually limitless potential of tetrazole compounds in various fields [54,55,56,57]. They have been successfully applied in pharmaceutical products as a potential replacement for *cis*-peptide binding. In addition, they are used as components in explosives, ligands in coordination chemistry, and precursors in preparing a diversified selection of heterocyclic compounds [58,59]. Considerable advancement was achieved by Wang et al. [60] by describing the synthesis, antiproliferative, tubulin polymerization, analysis of immunofluorescence staining, and cell cycle analysis of new tetrazole derivatives, **57**. Among the compounds synthesized, **57a** showed significant activity against SGC-7901, A549, and HeLa cell lines. The SAR detailed that the insertion of substituent into the ortho-position of the ring attached to the nitrogen atom of the triazole ring significantly improved the antiproliferative activity. The compounds bearing 3,4-dimethoxyl showed significant anticancer activities. The tubulin polymerization result showed that **57a** disrupts the microtubule network, arrests the cell cycle at the G2/M phase, and induces dose- and time-dependent apoptosis. Ulgheri and co-workers [61] reported designing and synthesizing a new class of active non-peptidomimetic and non-covalent caspase-1 inhibitors. Compound **58a** was identified to inhibit IL-1β release in activated macrophages in the low µM range, which corroborates the activities observed for the known covalent inhibitors. Due to the extensive application of altered nucleobases for cancer treatment as a PDE3 inhibitor. Shekouhy et al. [62] presented the synthesis, PDE3 and anticancer properties of some novel nucleobases/tetrazole hybrids using cilostazol as the core structure. Compounds **59a**, **59b** and **59c** are more strong inhibitors of PDE3A than cilostazol, and compound **59b** was observed as being the most effective PDE3A inhibitor (see Figure 12).

Additionally, the compounds **59a**, **59b** and **59c** showed significant inhibitory activity against HeLa (IC_50_ = 27.94 ± 0.36 µM) and MCF-7 (IC_50_ = 49.22 ± 1.01 μM) cancer cell lines. The presence of two purine-like nucleobases led to the strongest inhibitory effect against the PDE3A, while the insertion of a pyrimidine-like nucleobase also led to an enhanced inhibitory effect against the PDE3A and cytotoxicity activity against the HeLa and MCF-7 cell lines. Rashidipour et al. [63] reported the effectiveness of **60** on SKBR-3 cell proliferation as being similar to that of cisplatin, and the DNA-binding assay uncovered the ability of the compound to bind to DNA and alter its structure (see Figure 12).

### 2.5. Imidazole/Benzimidazole

**Figure 13:**
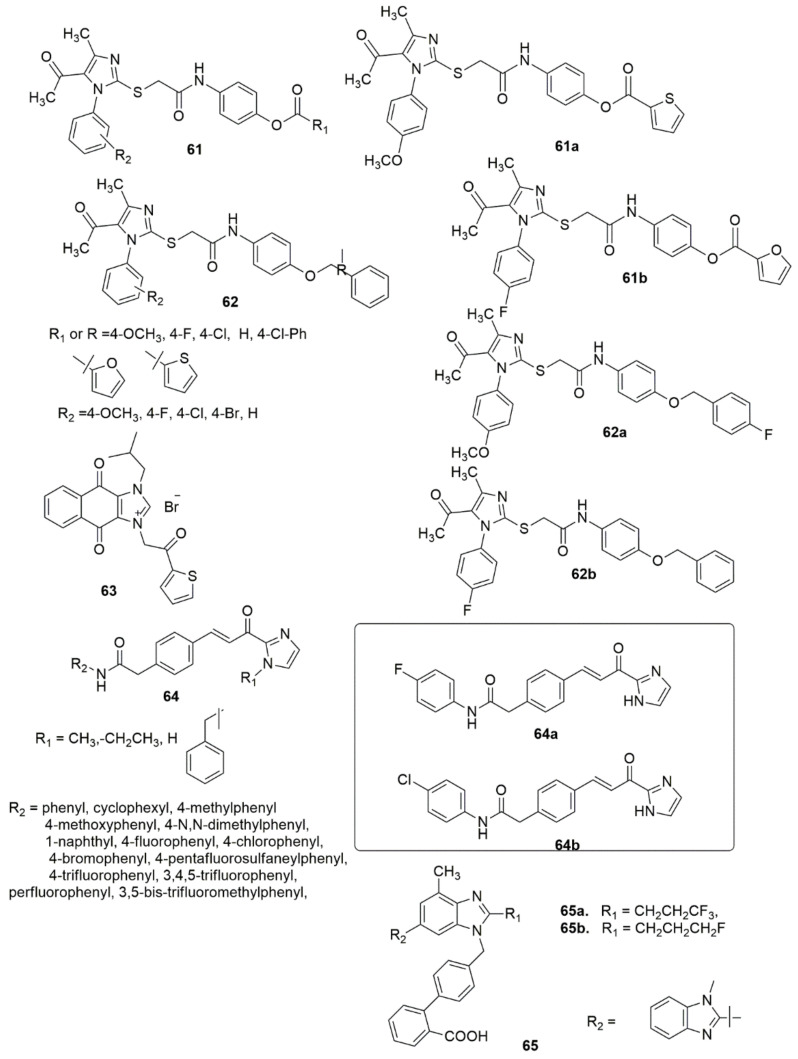
Analogues of imidazole with promising biological activities.

The development of synthetic pathways towards functionalized imidazole and benzimidazole is highly valued in both the chemical industry and academia [64]. Many synthetic molecules featuring imidazole or benzimidazole units have interesting biological activity and are valuable drug candidates. The stability, reactivity, basicity, amphoteric, and highly polar properties of imidazole and benzimidazole have been widely studied [65]. The potency and widespread applicability of the imidazole and benzimidazole pharmacophore may be attributed to their hydrogen binding donor acceptance ability. These compounds also have a strong affinity for metals found in many active proteins, enzymes, and receptor sites. As a result, many imidazole and benzimidazole derivatives have been reported with a broad spectrum of pharmacological effects. A library of new imidazole derivatives **61**, **62** have been prepared and evaluated for their biological activity [66]. Most of the examined compounds have significant inhibitory activities. Compound **61a** (MIC = 62.5, 100, 100 μg/mL) displayed broad-spectrum antibacterial activity against all ESBL, VRE, and MRSA strains, respectively, while **62a** (MIC = 25 μg/mL) showed excellent activity against the ESBL strain. All the examined compounds demonstrated lower activity than the standard drug to inhibit H37Rv strains. The in vitro antimalarial activity against *Plasmodium falciparum* showed that the analogues **61b** (IC_50_= 0.36 μg/mL), **61a**, and **62b** (IC_50_ = 0.45 μg/mL), exhibited moderate activity compared with the reference drug quinine (0.268 μg/mL). Forty novel naphthoquinone phenacylimidazolium derivatives were synthesized and subsequently evaluated for their antitumor activities against three human cancer cell lines [67]. Compound **63** exhibited remarkable activity against the MCF-7 cell line (IC_50_ = 50 nM) and 256-fold selectivity against normal cells. Furthermore, compound **63** was found to induce apoptosis, activate the pro-apoptotic protein caspase-3, and inhibit survivin expression. Al-Hamashi et al. [68] designed, synthesized, and described a new antimitotic agent class that modulates tubulin polymerization. All the compounds inhibited the growth of HCT 116 cells with GI50 values. The olefin moiety in the linker was crucial for the cytotoxic activity. Hydrogenation of this double bond or conversion to a cyclopropyl moiety obliterated the antiproliferative activity. Substitution of aniline moiety with a cyclohexyl group or a bulky naphthyl moiety lowered the antiproliferative activity, while the substitution of halogen atoms or a trifluoromethyl group at the *para*-position improved the inhibitory activity. Compound **64a** inhibited HDAC1, 2 and 3, while **64a** and **64b** had no effect on SMC3 acetylation. Compound **64a** further destabilized microtubules and accelerated depolymerization. A series of new fluoro-substituted benzimidazole hybrids were designed, synthesized and pharmacologically evaluated [69]. The new compounds were exposed to biological evaluation for their impacts on systolic blood pressure (SBP) and diastolic blood pressure (DBP) in spontaneously hypertensive rats. Of the compounds examined, **65a** and **65b** reduced blood pressure more effectively and had higher and more enduring antihypertensive effects than losartan and telmisartan at the same dose (see Figure 13).

**Figure 14:**
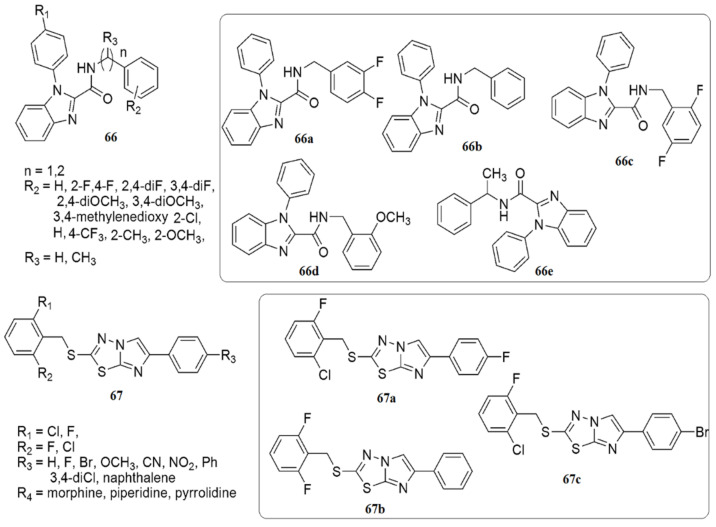
Structures of analogues of imidazole with promising biological activities.

Twenty-seven new compounds have been prepared via the NH_4_Cl-catalyzed amidation of ethyl benzo[d]imidazole-2-carboxylates [70]. The synthesized compounds were evaluated for antituberculosis (anti-TB) activity using the H37Rv strain. Thirteen compounds among the evaluated compounds had a superior MIC (0.78–6.25 μg/mL) to the standard drugs and were non-cytotoxic (<50% inhibition against RAW 264.7 cell lines at 50 μg/mL). The SARs indicated that the presence of lipophilic benzylamine improved the activity eight-fold in **66b** (MIC 1.56 μg/mL), while as a result of the integration of halogens, namely fluorine and chlorine, at the *ortho* position of the phenyl ring, the inhibitory activity decreased. Among the derivatives with disubstituted halogens, compound **66a** (MIC of 0.78 μg/mL) with 3,4-difluoro substituents demonstrated the highest activity. A four-fold reduction in anti-TB activity has been observed with the integration of the electron-withdrawing 4-trifluoromethyl group, whereas the presence of methyl at the *ortho* position reduced the activity due to the *ortho* steric clash. However, the activity increased upon incorporating the methoxy group at the same second position. In summary, **66a** exhibited the superior anti-tubercular potential with an MIC of 0.78 μg/mL (2.15 μM), followed by **66b**, **66c**, **66d** and **66e** with an MIC of 1.56 μg/mL (see Figure 14).

Askin et al. [71] investigated the synthesis, characterization, biological activity, and cytotoxic effects of imidazo[2,1-*b*][1,3,4]thiadiazole derivatives **67**. The novel imidazo[2,1-*b*][1,3,4]thiadiazole derivatives were tested for their ability to inhibit the ubiquitous cytosolic *h*CA I and *h*CA II isozymes and the cholinergic enzyme AChE. All the tested compounds demonstrated low nanomolar inhibitory activity against *h*CA I, *h*CA II, and AChE (*K*Is were 23.44–105.50, 10.32–104.70, and 20.52–54.06 nM, respectively). Moreover, compound **67a** inhibits *h*CA I up to 18-fold compared to acetazolamide, while compound **67b** has a five-fold selectivity towards *h*CA II. **67a**, **67b** and **67c** were the most potent inhibitors of *h*CA I and II isoforms, AChE, and non-toxic agents against the L929 mouse fibroblast cell line at their effective concentrations on target enzymes (see Figure 14).

**Figure 15:**
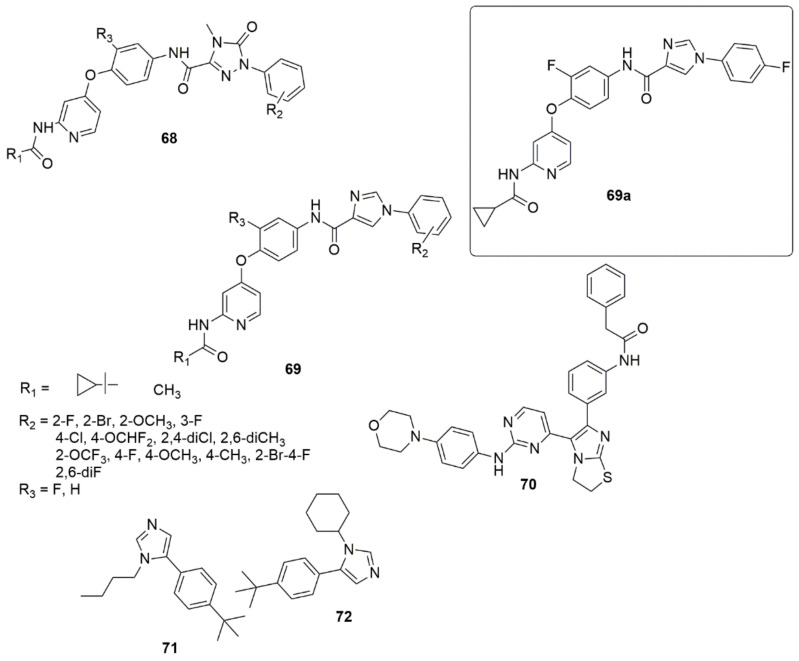
Chemical structures of imidazole hybrids with significant biological activities.

Twenty-six novel 4-phenoxypyridine bearing imidazole-4-carboxamide **68** and 4-methyl-5-oxo-4,5-dihydro-1,2,4-triazole-3-carboxamide **69** hybrids were designed, synthesized, and investigated for pharmacological activities [72]. All the newly synthesized target compounds were evaluated for their in vitro inhibitory activity toward c-Met kinase using a mobility shift assay. **69a** demonstrated the best activity with an IC_50_ value of 0.012 μM. The introduction of a fluorine atom on the phenoxy moiety was crucial for c-Met kinase effective activities for the two series of compounds. Based on an antiproliferative assay, compound **69a** showed remarkable proliferation reduction effects against MKN-45, A549 and H460 cell lines with IC_50_ values of 0.64, 1.92 and 2.68 μM, respectively. Additionally, compound **69a** strongly inhibited A549 cell motility. The results of a colony formation assay indicated that **69a** suppressed the colony formation and prevented the unobstructed increase of A549 cells, and induced apoptosis in MKN-45, A549, and H460 cells, in a concentration-dependent manner (see Figure 15).

Compound **70** has displayed promising activity with an IC_50_ value of 52 nM against IGF1R and an IC_50_ value of 35.5 nM against EGFR with an acceptable PK profile [73]. Compounds **71** and **72** both exhibited activity against HIV-1 in LEDGF/p75 contact, while **71** displayed a MIC value of 15.6 μg/mL against *S. aureus*, and **72** displayed a comparable MIC value against *B. cereus* [74] (see Figure 15).

**Figure 16:**
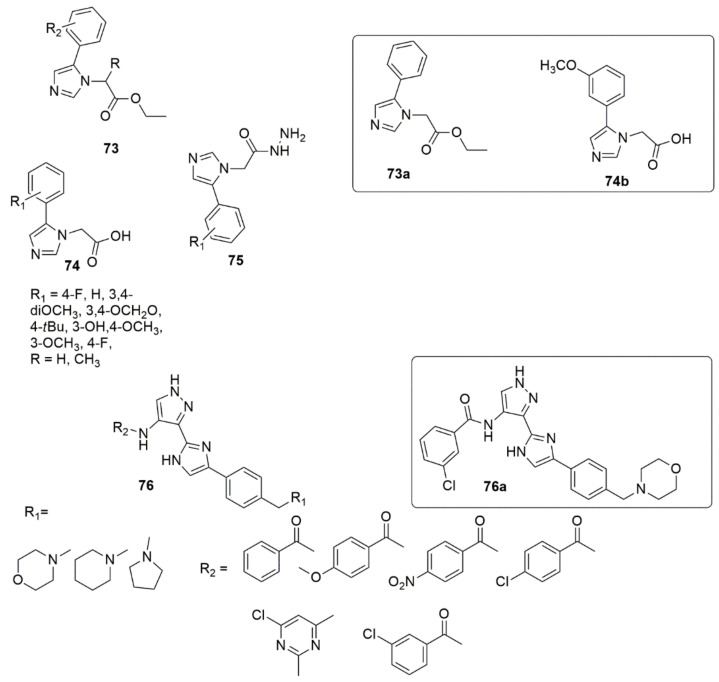
A new library of 2-(5-aryl-1*H*-imidazol-1-yl) compounds with significant biological activities.

A new library of 2-(5-aryl-1H-imidazol-1-yl) compounds **73**, **74**, **75** were designed, synthesized, and evaluated for their inhibitory activity against the HIV-1 Vpu and BST-2 protein interaction [75]. The results of the AlphaScreen™ assay showed that **73a** and **74b** displayed IC_50_ values of 11.6 ± 1.1, and 17.6 ± 0.9 µM, respectively, in a dose–response profile, whereas in cytotoxicity and antiviral assays, **73a** displayed significant activity with an EC50 value of 6.3 ± 0.7 µM at non-toxic concentrations (CC50 = 184.5 ± 0.8 µM), while compound **74b** exhibited an EC50 of 157.5 ± 1.2 µM (CC50 = 159.5 ± 0.9 µM). Thus, compound **73a** was identified as a potential inhibitor of HIV-1 Vpu and host BST-2 protein. A series of new 3-(4-phenyl-1H-imidazol-2-yl)-1H-pyrazole derivatives were designed and synthesized as JAK 2/3 and Aurora A/B kinase multi-target inhibitors by Zheng et al. [76]. Many of the compounds examined showed good inhibitory activity against JAK2/3 and Aurora A/B (with IC_50_ values ranging from 0.008 to 2.52 µM). Of all the compounds evaluated, **76a** remarkably decreased the toxic effect on normal human cells, more so than JAK 2/3 and the Aurora A/B kinase multi-target kinase inhibitor (AT9832). Compound **76a** downregulated the phosphorylation of STAT3, STAT5, Aurora A, and Aurora B in K562 and HCT116 cells. This potent compound induced cell cycle arrest in the G2 phase. Notably, the SAR showed that derivatives bearing a morpholine ring at the side chain exhibited superior antiproliferation activity compared to derivatives bearing a piperidine ring. Additionally, the presence of Cl, OCH_3_, and NO_2_ groups at the benzene ring enhanced the proliferative inhibition. However, compounds bearing electron-withdrawing groups such as Cl and NO_2_ displayed slightly higher K562 proliferative inhibition compared to the compounds containing an electron-donating group (OCH_3_) (see Figure 16).

**Figure 17:**
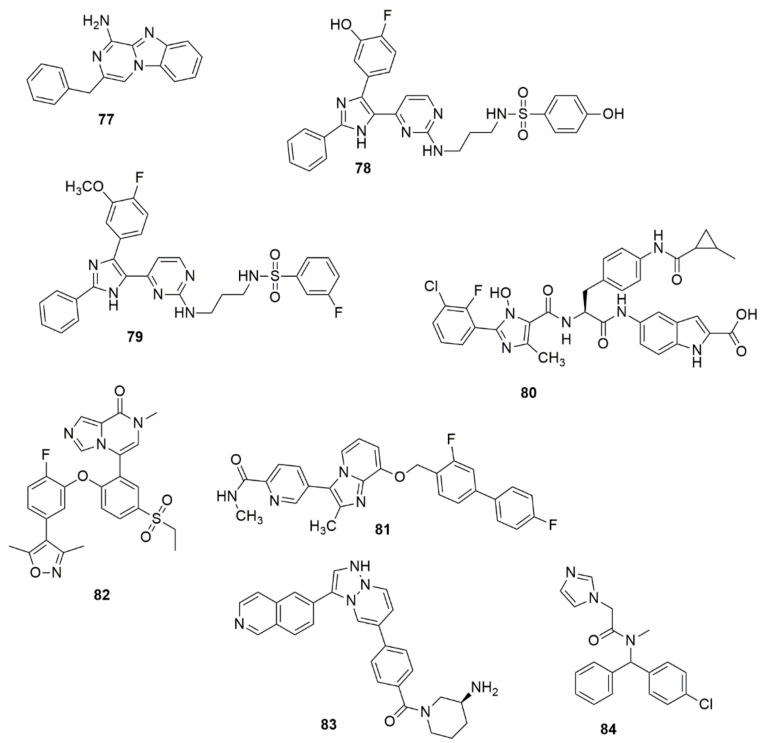
Structures of analogues of imidazole molecule with promising biological activities.

Reddy and co-workers designed and synthesized tricyclic benzimidazo[1,2-a]pyrazin-1-amine derivatives and subsequently investigated their antagonism in the adenosine A2A AR pathway [77]. Compound **77** showed potent binding affinity to A2A AR (IC_50_, 9.2 nM), good selectivity against A1 AR (A2A/A1 80-fold) and high potency in cAMP (IC50 31.0 nM) functional and IL-2 (EC50 = 164.6 nM) production assays. A series of dual imidazole-5-yl pyrimidine inhibitors BRAFV600E/p38a were developed and synthesized to overcome resistance to BRAFV600E inhibitors in BRAFV600E metastatic melanoma patients [78]. Among the examined compounds, **78** exhibited superior dual inhibition with IC_50_ values of 2.49 and 85 nM against BRAFV600E and p38a, respectively. Compound **78** exhibited high inhibitory activity with an IC_50_ value of 96.3 nM in the TNF-α production assay. The antiproliferative activity of the targeted compounds was determined using the MTT cytotoxicity assay. Compound **79** showed excellent antiproliferative activity with an IC_50_ value of 0.9 µM. The compound was 11.11-fold more selective against LOX-IMVI melanoma cells than the IOSE-80PC normal cell line. Lei and co-workers have proposed that **80** could be a potential and promising agent for the treatment of thrombotic diseases [79]. Sekiola et al. [80] discovered compound **81** as an attractive candidate for AD treatments. Compound **117** showed high in vitro potency with brain exposure and displayed an unnoticeable inhibition of cytochrome p450 enzymes. Concentrations of Aß42 in the brain of rats were significantly reduced in vivo at a dose of 10 mg/kg, while the dose of 2 mg/kg of **81** for 8 days fully saved the cognitive deficits of AD model mice. Compound **82** has exhibited excellent inhibitory activities against BRD4(1) with an IC_50_ value of 0.035 μM [81]. **82** successfully inhibited the proliferation of pancreatic cancer cells BxPC3. Compound **82** also arrested prostate cancer cells in the G0/G1 phase, induced cell apoptosis by regulating the expression of apoptotic proteins and demonstrated effective in vivo antitumor activity by inducing the apoptosis of tumor cells. Bu et al. [82] proposed compound **83** as potential MNK1/2 inhibitor [82]. Compound **84** has been shown to be a potential starting point for the development of a lead molecule used for the treatment of leukemia and glioblastoma (see Figure 17).

## 3. Six-Membered Heterocyclic Compounds

### 3.1. Pyrimidine

**Figure 18:**
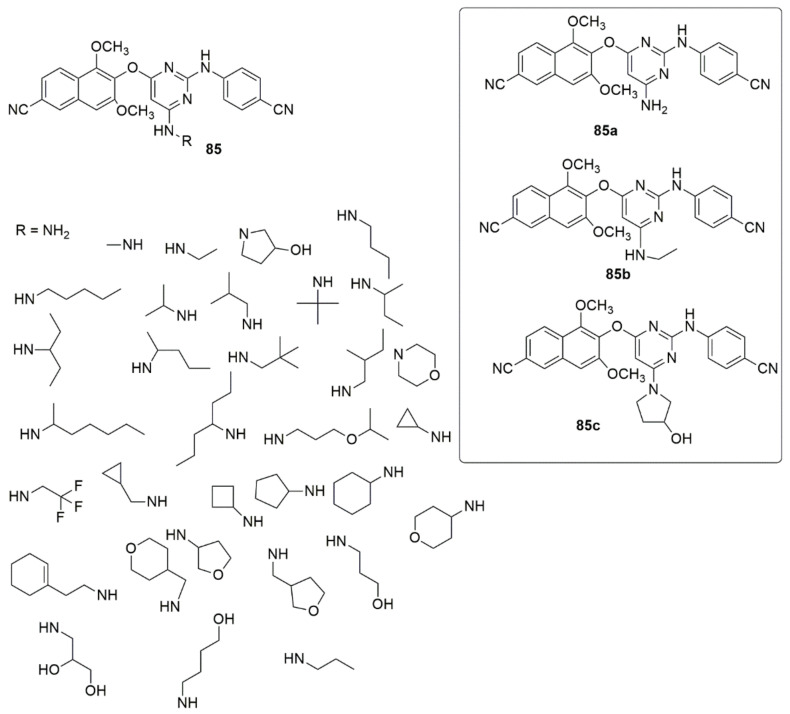
Structures of analogues of pyrimidine with promising biological activities.

**Figure 19:**
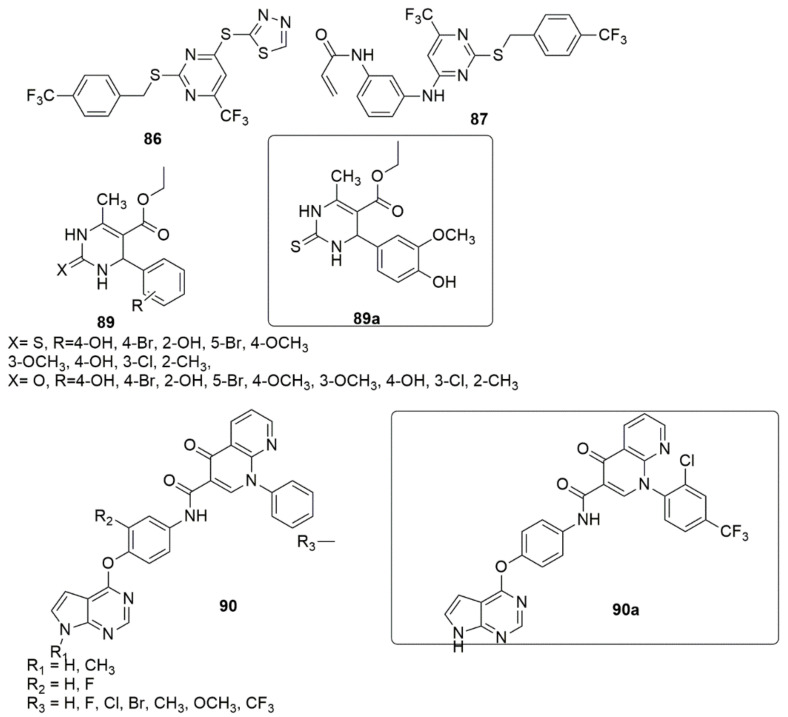
Structures of analogues of pyrimidine molecules with promising biological activities.

Pyrimidine is a privileged structure featuring two nitrogen atoms in its aromatic ring, positioned on the first and third carbon in the ring. The pyrimidine and derivatives include cytosine, thymine, uracil, thiamine, and alloxan. Many efforts were made to design and optimize pyrimidine derivatives since they are integral components of both DNA and RNA, and they revealed numerous novel pyrimidine derivatives with potent biological activities. A novel series of naphthyl-diarylpyrimidine (DAPY) **85** hybrids targeting the entry channel of non-nucleoside reverse transcriptase inhibitors binding pocket (NNIBP) were synthesized centered on the structure-based design strategy by Jin et al. [83]. The tested compounds displayed useful activities against the wild-type (WT) HIV-1 strain (EC50 values ranging from 0.0054 to 1.54 µM). The SAR analysis showed that cyclopropyl insertion improved antiviral activity with an EC50 of 0.020 µM. However, the inhibitory activity was decreased with the increased ring chains, indicating complexity for the wings to pass through the channel. Meanwhile, unsaturated alicyclic amines, namely, 2-(1-cyclohexenyl) ethylamine, decreased the anti-HIV activity (0.33 µM). Additionally, no change was observed when the cycloaliphatic groups were replaced with the oxygen-bearing heterocycles. The aminotetrahydrofuran derivative showed superior activity to the aminotetrahydropyran derivative, thus indicating that the five-membered ring is more appropriate than the six-membered ring to enter the channel. Compound **85a** showed excellent activity against HIV-1 (IIIB) with an EC50 value of 5 nM, equipotent to efavirenz (EC50 = 0.0043 µM), but less potent than etravirine (EC50 = 0.0037 µM). Moreover, compound **85b** bearing an ethylamine group has been found to have an EC50 of 0.0091 µM, with lower cytotoxicity (CC50 > 125 µM) and the highest selectivity index (SI > 30488), superior to EFV (SI > 1463) and ETR (SI > 1212). Compound **85c** showed inhibitory activity of EC50 = 0.011 µM, and a high selectivity index greater than 22,846. The HIV-2 analysis showed that many compounds were inactive against HIV-2 replication (see Figure 18). Fifty-two novel 2,4-substituted pyrimidine derivatives were synthesized, and the anti-proliferative activities of the targeted compounds against PC-3, MGC-803, MCF-7, and H1975 cells lines were evaluated using an MTT assay [84]. Compound **86** emerged as the most promising anti-proliferative agent of all the targeted compounds. It exhibited the best bioactivity against H1975 (IC_50_ = 2.27 μM), which is better than the standard drug 5-Fluorouracil with an IC_50_ of 9.37 μM, as well as compound **87** (IC_50_ = 4.77 μM). Compound **86** substantially inhibited the migration and colony formation of H1975 cells and induced cell cycle arrest at the G2/M phase. The molecular docking studies showed that compound **86** interacted with Val726 by a π-H interaction, while the 2-mercapto-1,3,4-thiadiazole group established hydrogen bond interactions with Arg841, Asn842, Phe723, and Gly724, respectively (see Figure 19).

A series of tetrahydropyrimidinone/thione hybrids **89** were synthesized using the Biginelli three-component reaction and subsequently evaluated their antiviral activity against replication of HIV-193IN101 (Subtype C) [85]. Compound **89a** displayed the best activity with an IC_50_ value of 2.2 μM. The derivatives containing a sulfur atom (IC_50_ values < 5 μM) in the pyrimidine ring showed better activity compared to the derivatives containing an oxygen atom (IC_50_ values and >5 μM). Compound **89a** formed a hydrogen bond with Asp368gp120, a crucial residue for CD4 binding during viral entry.

A series of pyrrolo[2,3-d]pyrimidine derivatives containing 1,8-naphthyridine-4-one analogs **90** were designed, synthesized, and their cytotoxic potency against three cancer cell lines (A549, Hela, and MCF-7) was examined [86]. Compound **90a** emerged as the most potent compound with IC_50_ values of 0.66, 0.38 and, 0.44 µM against A549, HeLa and MCF-7 cell lines, respectively. Notably, compound **90a** had a better apoptosis-causing effect than cabozantinib against A549 cells. In the enzymatic assay, the active compound **90a** showed selectivity on c-Met over six other tyrosine kinases. The SAR evaluation indicated that introducing a hydrogen atom (R_1_ group) improved the inhibitory activity compared to methyl group substituents. The cytotoxicity activity of the compounds also depends on the nature of the substituents on the R_3_. For example, the introduction of an electron-donating group at the 4-position of the terminal benzene ring reduced the activity. The insertion of double electron-withdrawing groups (double-EWGs) to R_3_ on the terminal phenyl ring affects the activity more positively than single electron-withdrawing groups (single-EWGs) (see Figure 19).

Wang et al. [87] have described the synthesis of 2,4-diamino pyrimidine derivatives **91** and **92**. Subsequently, the biological activities of the designed compounds were evaluated in vitro and in vivo. Most tested compounds displayed good anti-FAK activity, anticancer, and angiogenesis inhibitory effects. The SAR of benzylamine derivatives **91** was detailed. The introduction of halogens (F, Cl, Br) in the benzyl group’s *ortho* or *meta* position maintained the enzymes’ inhibitory activities. The insertion of a chlorine atom at the *ortho* position strongly inhibited FAK activity (**91a**, IC_50_ = 2.75 nM). However, the insertion of halogens at the para position affected the inhibitory effect. The compound with a hydrophobic methoxy group in the *ortho* position also demonstrated excellent inhibitory activity (IC_50_ = 5.73nM), while the inhibitory effect of the FAK enzyme was reduced by five-fold when the methoxy group moved to the *para* position. The SAR of the aniline derivatives showed that methoxy group substituent at the ortho position emerged as the most excellent FAK inhibitor (**92a**, IC_50_ of 1.87 nM). The activity reduced significantly when the methoxy group was shifted to the *meta* or *para* position, with IC_50_ values of 2321 and 55.63 nM, respectively. It was noted that the insertion of substituents to the *ortho* position positively influenced the FAK inhibition compared to meta and para positions, and the presence of the hydrophobic group was more favored for FAK inhibitory activity.

**Figure 20:**
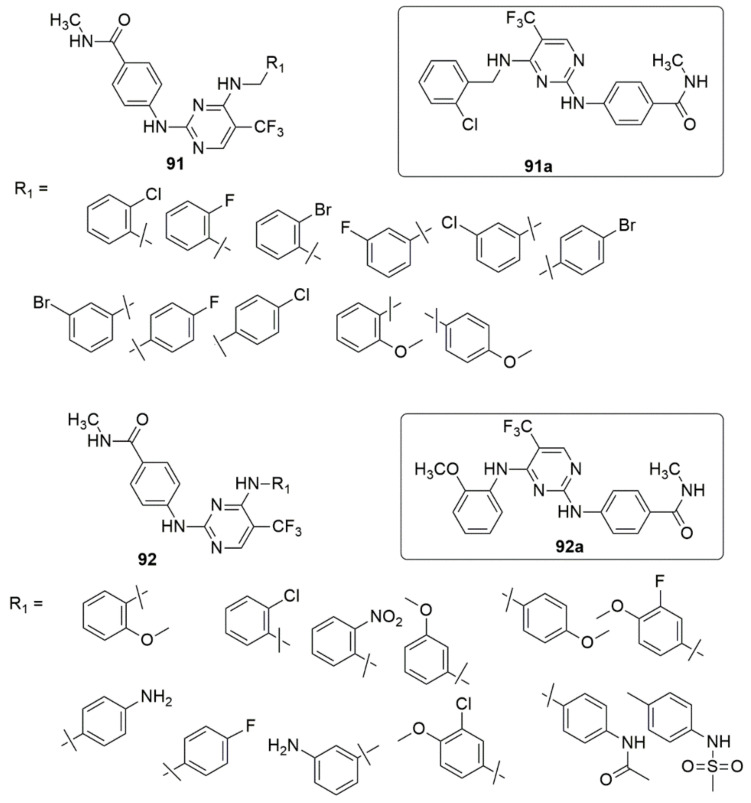
Analogues of 2,4-diamino pyrimidine derivatives with promising anti-FAK activity, anticancer, and angiogenesis inhibitory effect.

Additionally, the most effective compounds **91a** and **92a** against FAK-overexpressing pancreatic cancers PANC-1 and BxPC-3 cells suppressed the colony formation, migration, and invasion of PANC-1 cell dose-dependent. The two active compounds induced apoptosis of PANC-1 cells and arrested the cell cycle in the G2/M phase. Moreover, the compounds obstructed the FAK/PI3K/Akt signaling pathway and lessened cyclin D1 and Bcl-2 expression. Compounds **91a** and **92a** demonstrated remarkable antiproliferative properties. Also, the migration and tube formation of HUVECs in the anti-angiogenesis studies prevented the angiogenesis of zebrafish embryos in vivo (see Figure 20).

**Figure 21:**
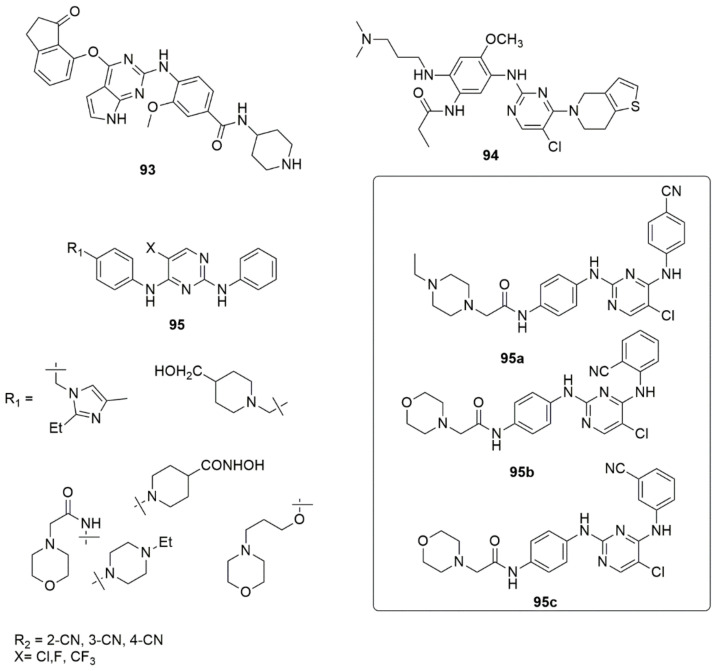
Analogues of pyrimidine molecule with promising biological activities.

Wei et al. [88] used scaffold-hopping strategies to design a series of pyrrolo[2,3-d]pyrimidine hybrids and evaluated their focal adhesion kinase (FAK) activity. Compound **93** (FAK, IC_50_ = 1.9 nM) showed the best FAK activity of all the evaluated compounds. Subsequently, compound **93** obstructed the phosphorylation of FAK signaling, cell passage, and invasion of PA-1 cells. Inhibition of tumor growth and metastasis in mouse models of ovarian cancer with insignificant side effects resulted in the oral administration of **93**. The molecular docking analysis showed that the formation of a hydrogen bond sandwiched between Cys502 and pyrimidin-2-amine was maintained (see Figure 21).

Li et al. [89] demonstrated that **94** displayed 42-fold selectivity for EGFRL858R/T790M over wild-type EGFRWT (IC_50_ = 4.0 nM). Its anti-proliferative activity was the best against H1975 cells (IC_50_ = 0.086 µM) and less potent against A549 and A431 cells (IC_50_ = 0.46 and 2.19 µM, respectively). The MOA of **94** was exerted by inhibiting cell migration and promoting apoptosis. The design, synthesis, and biological evaluation of cyano-substituted 2,4-diarylaminopyrimidines **95** as potent, non-covalent JAK3 inhibitors for the treatment of B-cell lymphoma have been reported by Wu et al. [90]. The 2,4-diarylaminopyrimidine derivatives featuring cyano substituents displayed potent inhibitory activities against JAK3, with IC_50_ values ranging from 20.66 to 334.3 nM. Compounds **95a** (IC_50_ = 22.86 nM), **95b** (IC_50_ = 20.66 nM), and **95c** (IC_50_ = 21.58 nM) demonstrated comparable potencies against JAK3 with tofacitinib (IC_50_ = 20.10 nM). It was found that both substitutions on the R_1_ and R_2_ influenced the inhibitory activities. The compound containing an *N*-ethylpiperazine substituent and cyano group **95a** successfully blocked ATP binding to JAK3. The substitution of the piperidin-4-ylmethanol group, which was meant to boost the hydrophilicity of the highly aromatic scaffold, exhibited a slight inhibition against JAK3, with IC_50_ values in the range of 40.20–75.95 nM. Meanwhile, insertion of aliphatic carbon as a spacer led to unfavorable anti-JAK3. Moreover, the derivatives bearing 4-piperidinehydroxamic acid substituents demonstrated poor inhibitory activities against JAK3, irrespective of the cyano group’s position or the substituent’s nature on the five-position of the pyrimidine ring. In contrast, the chlorine atom substituent enhanced the anti-JAK3 activity compared to either a fluorine or trifluoromethyl group. The most promising compound, **95a**, exhibited more-potent anti-proliferative activities against Ramos and Raji cells (IC_50_ values of 0.9255 and 1.405 μM, respectively), belonging to lymphoma B-cells. Compared to doxorubicin, it arrested the cells at the G2/M phase and influenced low toxicities against normal HBE, PBMC, and L-02 cells (see Figure 21)

**Figure 22:**
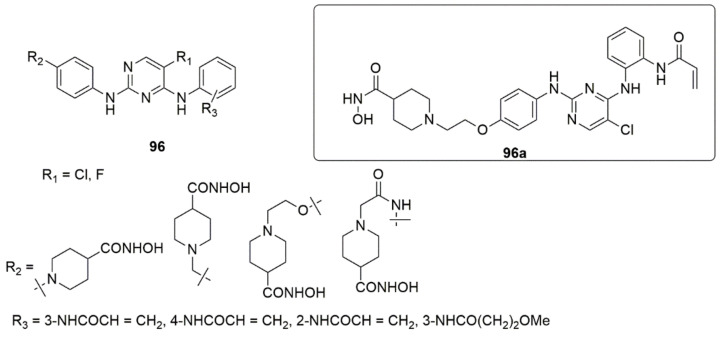
Series of 2,4-diarylaminopyrimidine tested for anti-proliferative activity.

To identify more effective and selective inhibitors against EGFR^T790M/L858R^, a series of 2,4-diarylaminopyrimidine derivatives **96** bearing hydrophilic isohydroxamic acid and carboxylic acid groups were designed, synthesized, and evaluated by in vitro kinase enzymatic assays [91]. The authors further evaluated the potent compound’s cellular activity assays and an in vivo xenograft mouse model. Of all the compounds tested, **96a** strongly inhibited EGFR^T790M/L858R^ mutated kinase with an IC_50_ value of 10.35 nM. It moderately suppressed the proliferation of H1975 cells transfected with the EGFR^T790M/L858R^ mutant with an IC_50_ value of 0.2113 μM compared to A431 (IC_50_ > 10μM). Additionally, **96a** displayed an excellent selectivity index (SI > 47.3) and weak inhibition against the proliferation of HBE (IC_50_ > 40 μM) and L-02 (IC_50_ = 3.058 μM) cells, respectively. Compound **96a** further induced apoptosis by arresting the H1975 cells in the G2/M phase and demonstrated anti-cancer efficacy in the H1975-driven xenograft mouse model in vivo. Notably, the introduction of an ethoxy piperidine linker enhanced the anti-proliferative activity. However, the replacement with acetamide lessened the activity against H1975. The 2,4-diarylaminopyrimidines derivatives containing esters and carboxylic acid repressed the proliferation of the H1975 and L-02 cell lines, while the insertion of hydroxamic acid groups improved the anti-proliferative activity (see Figure 22).

**Figure 23:**
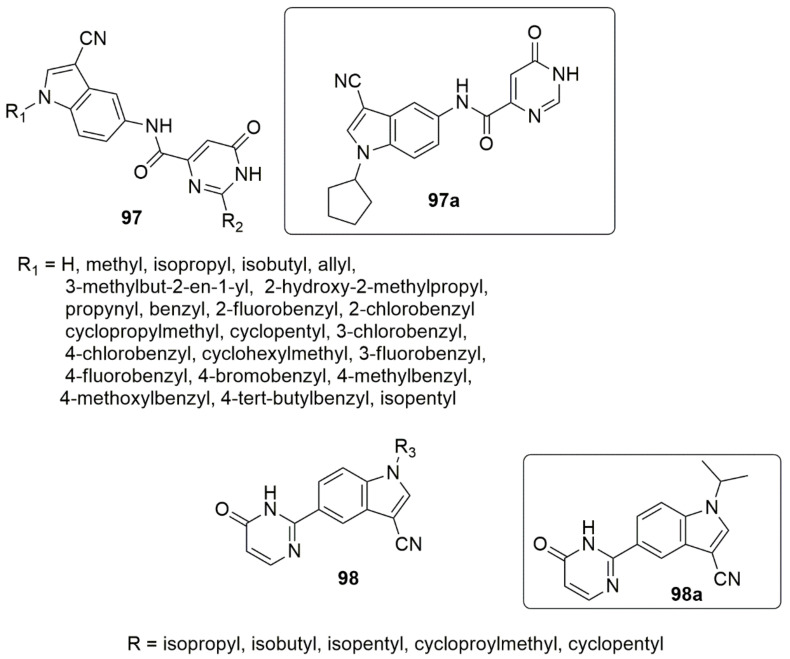
Chemical structures of pyrimidinone derivatives evaluated for XO inhibitory potency.

The fragment-based drug design (FBDD) approach was used for networking two possible fragments, specifically pyrimidinone and 3-cyano indole, with an amide bond to project pyrimidinone derivatives **97** and **98**, respectively [92]. The inhibitory activity of xanthine oxidase (XO) in vitro of the synthesized compounds was evaluated by spectrophotometry. Compound **97a** displayed an XO inhibitory potency with an IC_50_ value of 0.16 µM, 52.3-fold superior to allopurinol (IC_50_ = 8.37 µM). Meanwhile **98a** (IC_50_ = 0.085 µM), was 98.5-fold superior to allopurinol (IC_50_ = 8.37 µM), and hence was observed as the most excellent compound. These indicated the crucial effect of amide and single bonds as a spacer between pyrimidinone and the indole ring, which enhances potent nonpurine XO inhibitors. The results of the SAR showed that hydrophobic substitutions at position one of the indole fractions were essential for in vitro inhibitory power against XO. Furthermore, enzymatic kinetics studies have suggested that compounds **97a** and **98a** act as mixed-type non-purine XO inhibitors. The results of experimentation with hypouricemic activity in vivo demonstrated that **97a** and **98b** were potential and effective agents for treating hyperuricemia when compared to the sample group at 1 h after oral administration of the medication at 10 mg/kg (see Figure 23).

**Figure 24:**
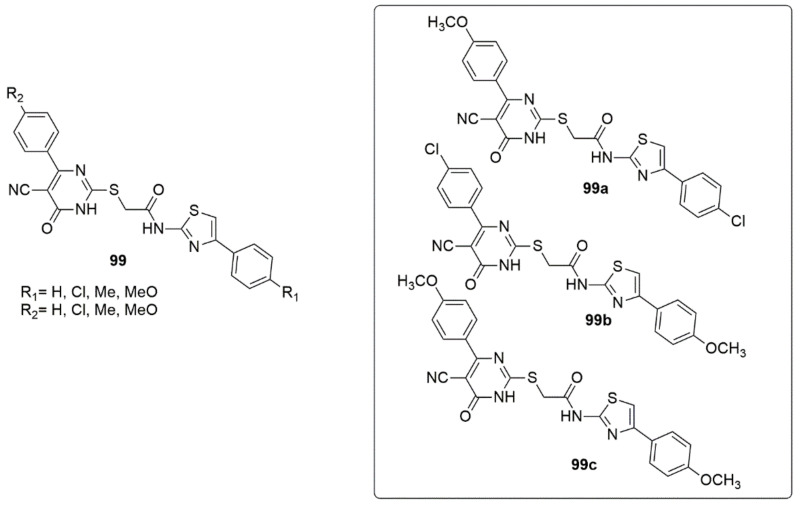
New series of pyrimidine-5-carbonitrile hybrids with 2-amino-4-aryl-1,3-thiazole using acetamide group spacer.

Synthesis of a new series of pyrimidine-5-carbonitrile hybrids with 2-amino-4-aryl-1,3-thiazole using acetamide group spacer as anti-inflammatory EGFR inhibitors has been reported [93]. In vitro COX-1/COX-2 and 15-LOX inhibitory activities of newly synthesized derivatives have been reported. Among the tested compounds, compounds with methoxy and chlorine groups demonstrated the most potent COX-2 inhibitory activity (**99c**, IC_50_ = 1.13 μM, SI = 8.21; **99b**, IC_50_ = 1.13 μM, SI = 7.84), although the activity was lower than the reference compound celecoxib (IC_50_ = 0.88; SI = 8.31). Compounds **99c** and **99b** were observed as 15-LOX inhibitors with IC_50_ values of 5.29 and 5.73 μM, respectively, compared to meclofenamate sodium (IC_50_ = 5.64 μM). The carrageenan-induced rat paw edema method was employed for the anti-inflammatory assay. The results indicated that compounds **99a**, **99b**, and **99c** exhibited respectively 94, 86, and 90% anti-inflammatory activity compared to meloxicam after 4 h. The active compounds were then subjected to ulcerogenic tests, and the compounds showed improved gastric safety profiles than meloxicam. In addition, a significant reduction in pro-inflammatory cytokines (PGE2, TNF-α, IL-6), iNO, MDA, and a desirable significant induction of TAC with no effect on either blood pressure or heart rate was detected. Compounds **99a**, **99b**, and **99c** had a superior inhibition among the tested compounds in anti-proliferative and EGFR inhibitory assays (see Figure 24).

**Figure 25:**
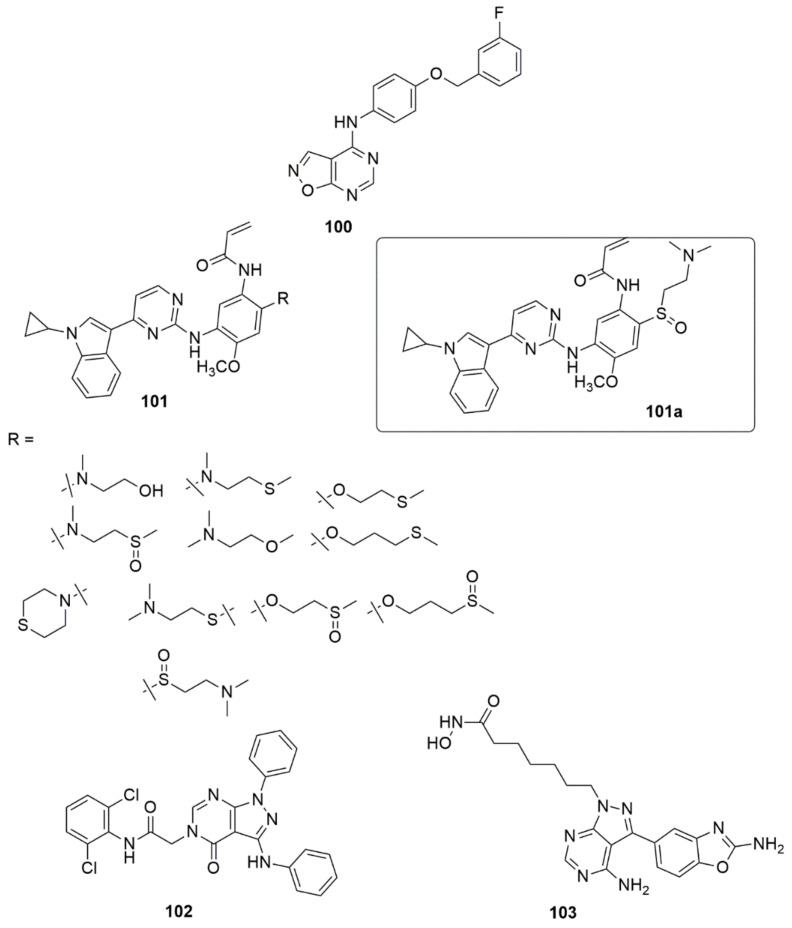
Chemical structures of pyrimidinone derivatives with promising biological activity.

Compound **100** has been reported as a promising anticancer agent with inhibitory growth effects of GI50 values of 0.0221 μM HCT-15 (colon), 0.0318 μM MDA-MB-435 (melanoma), 0.0263 μM SNB-75(CNS), and 0.0372 μM MCF7 (breast) [94]. Compound **100** induced apoptosis in BT-474 cancer cells, which could be a possible mechanism of action for compound **100**. Li et al. designed and synthesized a new series of osimertinib derivatives due to the aloofness of the *N*-methyl group of the indole ring in the osimertinib structure, thus releasing small quantities of metabolite and causing adverse effects during its application [95]. The compounds were evaluated for their biological activities. The derivatives bearing an indole *N*-cyclopropyl ring and sulfoxide side chain at the C-4 position of the aniline group exhibited excellent inhibitory activity against the H1975 and PC9 cells, with IC_50_ values of 0.008 and 0.004 µM, respectively. The potent compound **101a** (89.8, WT/TL) displayed a better selectivity than AZD9291 (84.0, WT/TL). Meanwhile, the SAR showed that the derivative bearing thioether (IC_50_ = 0.021 µM) was less potent than **101a**. This suggested that the presence of the sulfoxide group at the side chain directly connected to the phenyl ring increase the antiproliferative activity. Also, methylthio and thiomorpholine moieties at the end of the side chain showed reduced activity against the H1975 cells, while the compounds substituted with chlorinated pyrimidine rings exhibited a good antiproliferative effect against the H1975 cells. Compound **101a** demonstrated excellent inhibitory activity (IC_50_ = 0.26 µM) against the T790M/L858R double mutant EGFR kinase, superior to AZD9291 (IC_50_ = 3.40 µM). The xenograft model using bare mice revealed that **101a** showed powerful antitumor efficacy in vivo. In addition, **101a** may selectively inhibit tumors cells with low toxicity to normal human cells (see Figure 25).

Compound **102** was reported as a promising anticancer agent [96]. The compound was active against a panel of four cancer cell lines, HepG-2, MCF-7, HCT-116, and Hela, with IC_50_ values of 4.28, 5.18, 3.97, and 9.85 μM, respectively. Also, **102** (IC_50_ = 56.02 ± 1.38 μM) demonstrated better inhibition against EGFRWT compared with gefitinib as control drug (IC_50_ = 41.79 ± 1.07 μM) in the HTRF assay. Compound **102** further induced apoptosis and cell cycle arrest at Pre-G1 and G2/M phases. Zhai and co-workers [97] demonstrated that compound **103** exhibited excellent antiproliferative activity against HepG2 cells with an IC_50_ of 1.61 µM compared to the reference compounds (MLN0128, IC_50_ = 2.13 µM and SAHA, IC_50_ = 2.26 µM). Compound **103** showed significant selective cytotoxicity against HepG2 cells over normal human liver cells (L-O2) with a high SI value of 15.41. Also, **103** demonstrated significant inhibition of mTOR kinases and HDACs such as HDAC1, HDAC2, HDAC3, HDAC5, and HDAC8, in the in vitro enzyme inhibition assays. The active compound further induced apoptosis, arrested the cell cycle of HepG2 cells at the G0/G1 phase, and hampered HepG2 cell migration. It also displayed great metabolic stability in the rat liver microsome assay (see Figure 25).

**Figure 26:**
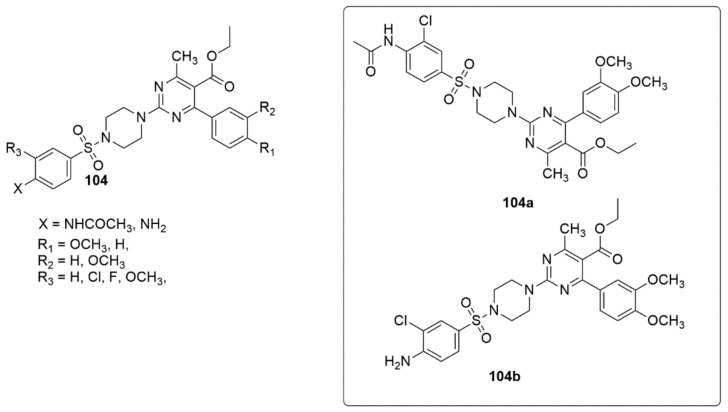
New series of phenylsulfonyl–pyrimidine carboxylate derivatives as dual inhibitors of AChE and BuChE.

Based on the pharmacophore amalgamation approach, a series of new phenylsulfonyl-pyrimidine carboxylate derivatives as dual inhibitors of AChE and BuChE were designed and synthesized [98]. Among the evaluated compounds, compounds **104a** and **104b** exhibited excellent inhibition against AChE, with an IC_50_ value of 47.33 ± 0.02, 51.36 ± 0.04, and BuChE with an IC_50_ value of 159.43 ± 0.72, 153.3 ± 0.74 nM. The activity of these compounds was influenced by the electron-donating (dimethoxy) and electron-withdrawing (Cl), which increased the interaction toward AChE. The presence of either an electron-donating or electron-withdrawing group at the substituting position led to moderate to good inhibitory activities. The MC65 neuroprotection assay revealed that **104a** and **104b** demonstrated neuroprotection at 80 µM while **104a** was estimated to be 99.41 in relation to tetracycline was a considerable neuroprotective agent compared to **104b** (see Figure 26).

### 3.2. Quinoline

Quinoline is a six-member fused nitrogen-containing compound. The uses of quinoline derivatives are rapidly spreading from anti-cancer drugs to almost every branch of medicinal chemistry. Including antimalarial, anti-bacterial, antifungal, anthelmintic, cardiotonic, anticonvulsant, anti-inflammatory, and analgesic activity. Quinoline derivatives represent many antiproliferative agents with DNA-intercalated cytotoxicity, causing interference with the replication process [99].

**Figure 27:**
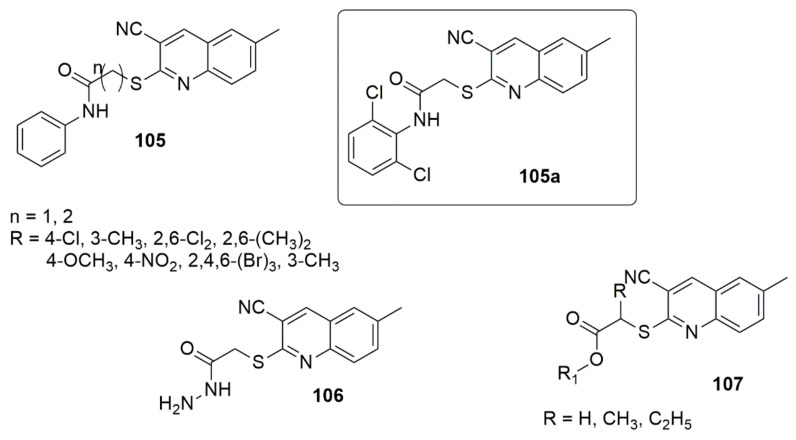
New series of quinoline derivatives with promising antimicrobial activity.

El-Shershaby et al. [100] designed a series of new compounds by employing modification fluoroquinolones and evaluated their activities against pathogenic bacterial and fungal strains. The tested compound (**105a**) with two substituted halogen atoms exhibited the highest potent antimicrobial activity. The observed MIC values were 0.98, 0.74, 3.98, 0.79, 3.80, and 0.66 μg/mL against *S. pneumoniae*, *B. subtilis*, *E. coli*, *A. fumigatus*, *S. racemosum*, and *G. candidum*, respectively. The in vitro activity against the *E. coli* DNA gyrase further demonstrated the considerable potent inhibitory activity of compound **105a** with an IC_50_ value of 3.39 μM. The SAR investigation showed that the derivatives bearing three bulky bromine atoms displayed a lower activity. At the same time, the elongation of the amide spacer **106** also showed a negative impact on antimicrobial activity. Furthermore, the compounds bearing an ester functional group **107** exhibited moderate to good activity (see Figure 27).

**Figure 28:**
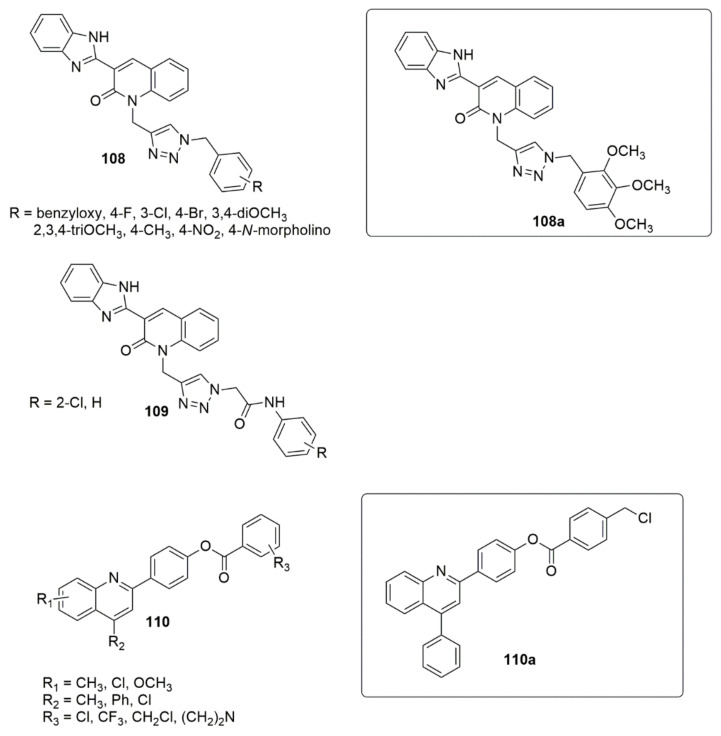
Chemical structures of quinoline derivatives with promising biological activities.

Gaikwad and co-workers’ [101] modeled 1,2,3-triazole containing a 2-oxo-quinolinebenzimidazole moiety by minimizing the pharmacophoric characteristics. The synthesized derivatives were screened against the NCI-60 cancer cell lines’ panel to evaluate their in vitro antiproliferative activity. Compound **108a** has promising anticancer activity on the central nervous system (CNS), leukemia, melanoma, ovarian, and breast cancer cell lines. Compound **108a** was further evaluated to verify apoptosis induction potential and the cytotoxic activity against the human breast cancer line (BT-474). The SAR detailed that the compounds bearing halogen substituents (R = 4-F and 3-Cl) exhibited good to moderate growth inhibition on the colon, melanoma, renal, and breast cancer cell lines. However, the Br derivative (R = 4-Br) lacked inhibitory activity. The trimethoxy substituent increases the antiproliferative activity, while the dimethoxy derivative exhibited a loss of potency. Besides, the *para*-methyl derivative exhibited better growth inhibition than the dimethoxy derivative. The *para*-morpholino derivative had a significant growth-inhibitory effect on renal, ovarian, prostate, and breast cancer cell lines. Meanwhile, in the second series of the synthesized compounds (**109**), the presence of a chlorine atom substituent significantly increased the inhibitory activity of the breast cancer cell lines. However, the removal of the chlorine atom yielded moderate growth inhibition. Preliminary screening studies based on the MTT assay revealed that compound **108a** had an excellent antiproliferative effect against human breast cancer cells, BT-474, with an IC_50_ value of 0.59 ± 0.01 µM. Compound **108a** further induced apoptosis on the BT-474 cell line. In the work of Karnik et al. [102], novel substituted quinoline derivatives were designed, synthesized, and evaluated as new L858R/T790M/C797S triple-mutant EGFR-TKIs. The anti-cancer activity of the synthesized derivatives was evaluated against HCC827, H1975 (L858R/T790M/C797S and L858R/T790M), A549, and HT-29 cell lines using an MTT assay. Most of the quinoline derivatives revealed a significant cytotoxic effect. Compound **110a** with a Cl group substituent (R_3_ = CH_2_Cl) on the quinoline ring displayed the most promising anticancer activity with an IC_50_ value of 1.91μM against EGFR kinase triple mutant. Besides, the compound displayed IC_50_ values of 0.0042, 3.82, and 3.67 μM against HCC827, A549, and HT 29, respectively. The placement of the phenyl ring (R_2_ = Ph) at position four of quinoline moiety yielded good anticancer activity. Nevertheless, the replacement of the phenyl ring with other substituents resulted in a decrease in activity. In addition, it was demonstrated that replacing the chloromethyl or trifluoromethyl group on the benzoate ring with NH_2_ decreased the activity. The most potent compound’s apoptosis and cell cycle progression analysis showed that compound **110a** induced early apoptosis and late apoptosis and arrested cell cycle at the G0/G1 phase (see Figure 28).

**Figure 29:**
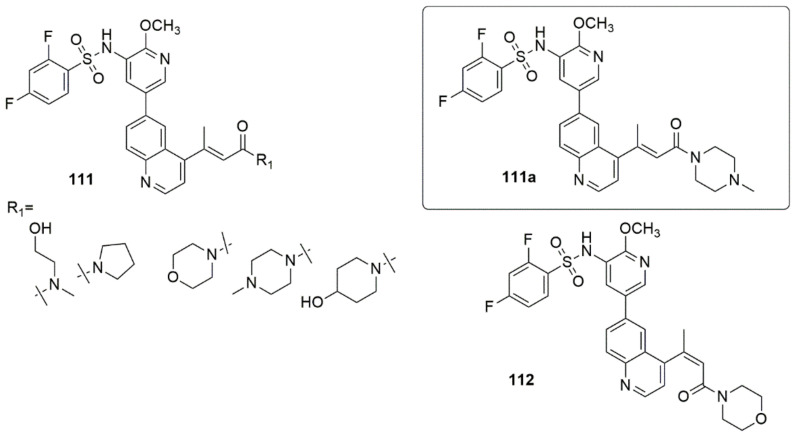
Chemical structures of 4-acrylamido-quinoline derivatives evaluated for PI3K/mTOR activity.

He et al. [103] synthesized effective 4-acrylamido-quinoline-derived PI3K/mTOR dual inhibitors with enhanced oral exposure and promising in vitro and in vivo potency [103]. All the target compounds were first assayed for their inhibitory activities against PI3Kα with the clinically probed GSK2126458 as the standard compound. Of all the compounds investigated, **111a** displayed the best inhibitory activity against PI3Kα with an IC_50_ value of 0.80 nM, comparable to that of GSK2126458. Additionally, compound **111a** exhibited the most engaging activity against the U87MG cell line with a GI50 value of 0.14 µM and effectively down-regulated the PI3K/Akt/mTOR pathway by obstructing PI3K and mTOR signaling. **111a** displayed potent inhibition against PI3Kα, PI3Kβ, PI3Kγ, PI3Kδ, and mTOR, with corresponding IC_50_ values of 0.80, 0.67, 1.30, 1.30, and 5.0 nM, respectively, which suggested that it was a potent pan-class I PI3Ks/mTOR dual inhibitor. The in vivo experimentation revealed that **111a** significantly improved oral exposure and had a strong therapeutic efficacy in a U87MG glioblastoma xenograft model. The PI3Ka inhibitory activity **111a**, *E* configuration was more active than the *Z*-configuration compound (**112**), suggesting that the *E* configuration carbon–carbon double bond improved the enzymatic activity (see Figure 29).

**Figure 30:**
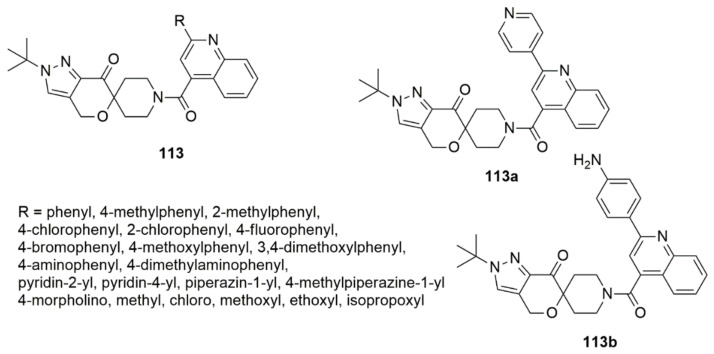
Chemical structures of spiroketopyrazole derivatives as novel acetyl-CoA carboxylase (ACC) inhibitors.

A series of spiroketopyrazole derivatives bearing quinoline moieties have been designed and synthesized as novel acetyl-CoA carboxylase (ACC) inhibitors [104]. The targeted compounds were examined for their biological activity toward the ACC1 enzyme and three different cancer cell lines, namely A549, HepG2, and MDA-MB-231. The biological evaluation showed that compound **113a** (R = 4-aminophenyl) exhibited the strongest enzyme inhibitory activity with an IC_50_ value of 1.29 nM. The pyridine-substituted derivatives (R = pyrindin-2-yl or pyridine-4-yl) exhibited equipotent activities with an IC_50_ values of 13.84 and 11.31 nM, respectively, superior to PF-1 [105] (IC_50_ = 17.28). The derivative with the phenyl-substituted compound (R = phenyl, IC_50_ = 6.00 nM) was two-fold lower than PF-1, suggesting the effectiveness of phenyl in inhibiting the enzymatic activity. However, the replacement of phenyl with electron-neutral (R = 4-CH_3_) or electron-rich (R = 4-OCH_3_, 3,4-OCH_3_, 4-NH_2_, 4-N(CH_3_)_2_) groups have negative impact on the ACC inhibitory activity. Compound **113b** displayed the most potent anti-proliferative activity against A549, HepG2, and MDA-MB-231 cells with corresponding IC_50_ values of 0.55, 0.38, and 1.65 nM, respectively. The pharmacological investigation revealed that compound **113b** lessens the intracellular malonyl-CoA and TG levels in a dose-dependent manner. The most active compound (**113b**) also significantly down-regulates cyclin D1 and CDK4 to disturb the cell cycle and up-regulates Bax, caspase-3, and PARP along with the destruction of Bcl-2 to induce apoptosis. Notably, the combination therapy involving **113b** and doxorubicin led to synergistic outcomes regarding the inhibition of HepG2 cell growth, indicating its ability to act in combination or in adjuvant therapies (see Figure 30).

### 3.3. Quinoxaline

**Figure 31:**
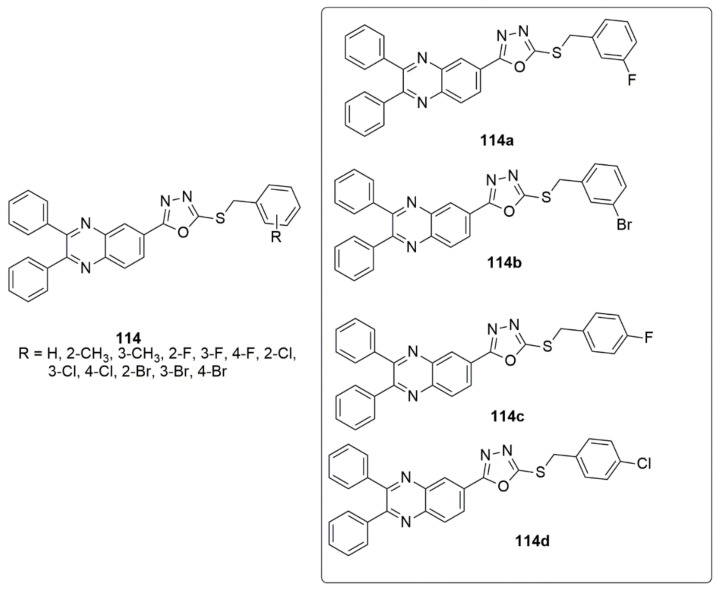
Chemical structures of new series of quinoxalin-1,3,4-oxadiazole derivatives with anti-Alzheimer activity.

In the continuation of the work of Mirzazadeh et al. in discovering new and effective inhibitors against metabolic enzymes, they reported the synthesis of new series of quinoxalin-1,3,4-oxadiazole (**114**) derivatives. They demonstrated their inhibitory effects against human carbonic anhydrase (hCA) isoenzymes I and II (carbonic anhydrases I and II), cholinesterase (ACHE and BCHE), and α-glucosidase [106]. All the targeted compounds displayed better enzymatic inhibitory activities than the standard inhibitors. Of all the tested derivatives, compound **114a** with IC_50_ values of 594.27 and 624.27 nM displayed approximately two-fold higher inhibitory activities than tacrine (IC_50_ = 1237.20 nM) against AChE and BChE, respectively. Compound **114b** (IC50 = 24.37 nM) showed 4.45-fold superior inhibitory activities than acarbose (IC_50_ = 1060.30 nM) in the α-glucosidase assay. Moreover, compounds **114c** and **114d** displayed 7.37-fold higher inhibitory activities than acetazolamide, against hCA I (IC_50_ value = 103.28 ± 9.38 and 109.37 ± 13.28 nM, respectively). Also, it was 6.8-fold superior to acetazolamide in II isoenzymes (IC_50_ values = 140.83 ± 32.04 and 128.83 ± 50.43 nM, respectively). Hence, the derivatives acting as cholinesterase inhibitors can be further considered as potential agents for the treatment of Alzheimer’s disease and other neurological disorders (see Figure 31).

**Figure 32:**
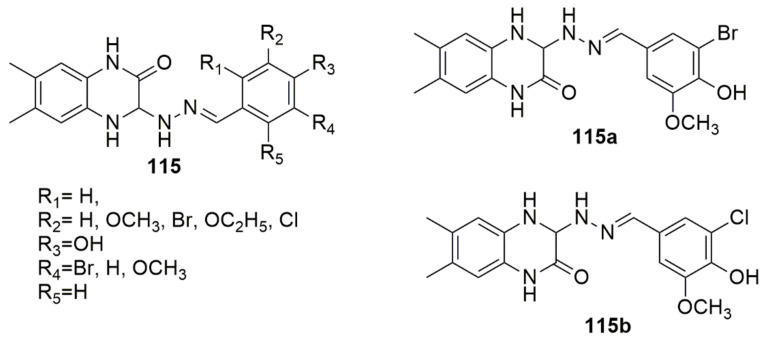
Chemical structures of new 6,7-dimethyl quinoxaline hybrids acting as GSK3β inhibitors.

Kumar and co-workers [107] designed and synthesized a series of new 6,7-dimethyl quinoxaline hybrids and subjected the synthesized compounds to kinase assay. Among the tested compounds, **115a** and **115b** are exceptionally selective towards GSK3β in kinase assays. Compound **115a** with a *p*-Br substituent in the aromatic ring was found to be the most potent, with an IC_50_ of 0.270 µM, while **115b** with a *p*-Cl derivative exhibited an IC_50_ of 0.390 μM; this could be due to the enhanced non-covalent interactions with the residues of the pivotal region Val135, with regard to molecular modeling studies (see Figure 32).

**Figure 33:**
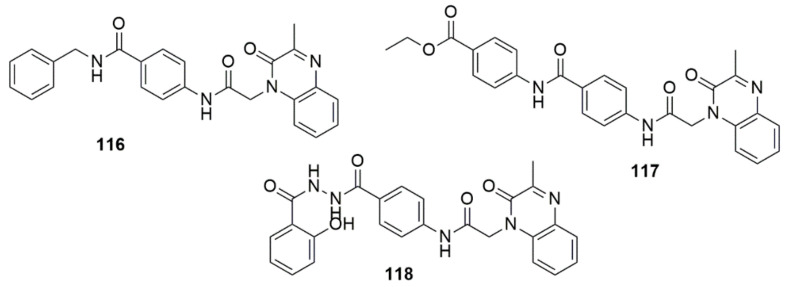
Chemical structures of quinoxaline-2(1*H*)-one derivatives with antiproliferative activity.

El-AdI et al. [108] unraveled quinoxaline-2(1*H*)-one derivatives (**116**, **117** and **118**) as influential compounds against HepG-2, HCT-116 and MCF-7 cell lines, respectively. Compound **116** (IC_50_ = 5.30, 2.20, 5.50 nM, respectively) was the most active compound compared to doxorubicin (IC_50_ = 8.28, 9.63, 7.67 μM) and sorafenib (IC_50_ = 7.31, 9.40, 7.21 μM). **117** demonstrated superior anti-proliferative activities compared to doxorubicin and sorafenib against HepG-2 and HCT-116. However, its activity was inferior in the MCF-7 cell line (IC_50_ = 8.72 μM). Additionally, compounds **116**, **1117** and **118** effectively impeded VEGFR-2 at IC_50_ values of 1.09, 1.49, and 1.19 μM, respectively, compared to the reference drug sorafenib (IC_50_ = 1.27 μM) (see Figure 33).

**Figure 34:**
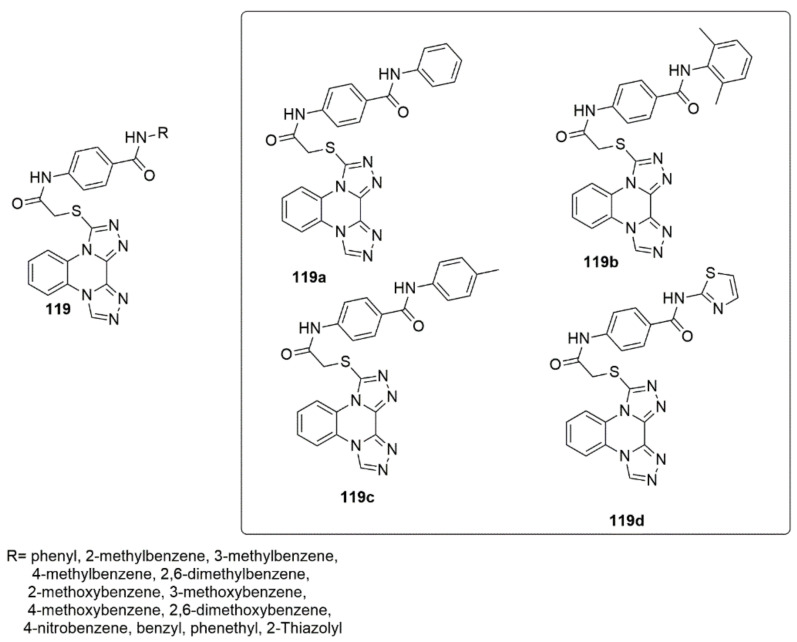
Chemical structures of new triazolo-quinoxaline scaffolds acting as VEGFR-2 inhibitors.

A new library of triazolo-quinoxaline scaffolds was designed, synthesized, and subjected to VEGFR-2 inhibitory activities. The targeted compounds were more selective toward HepG2 cells than MCF-7 cells [109]. Among the tested compounds **119a** (IC_50_ = 11.4 and 14.2 μM), **119b** (IC_50_ = 10.7 and 13.7 μM), **119c** (IC_50_ = 3.3 and 4.4 μM), and **119d** (IC_50_ = 8.7 and 11.3 μM) demonstrated high inhibitory activity against HepG2 and MCF-7, respectively. Compounds **119a**, **119b**, **119c**, and **119d** further successfully and effectively inhibited VEGFR-2 with IC_50_ values of 5.7, 6.7, 3.2, and 3.1 μM, respectively, compared to the standard compound (sorafenib, IC_50_ = 3.1 µM). Notably, the presence of 4-methoxybenezene drastically increased the VEGFR-2 inhibition. Moreover, the most potent compound, **119c**, arrested the cell cycle at the G2/M phase and induced apoptosis by 3.5-fold compared to the control. At 20 h, it upregulated caspase-3, caspase-9, and BAX by 2.07-, 1.72-, and 1.83-fold, respectively, and downregulated the Bcl-2 level by 1.92-fold (see Figure 34).

### 3.4. Purines

**Figure 35:**
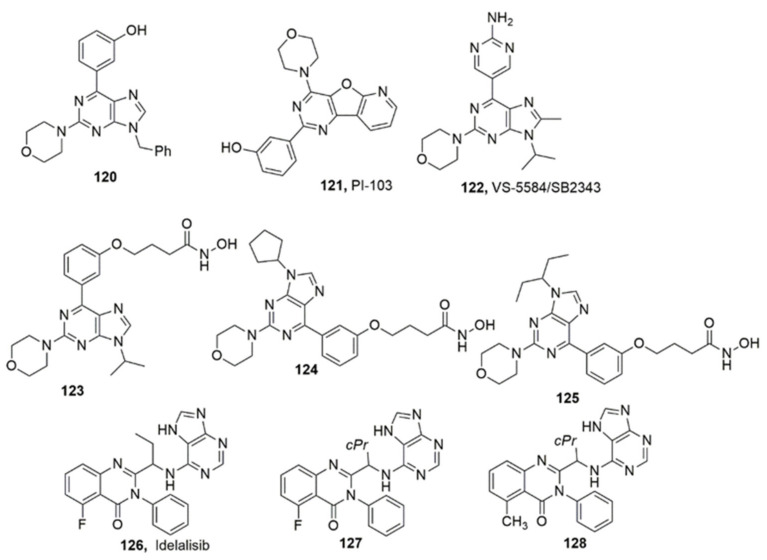
Chemical structures of purine derivatives with promising therapeutic effects.

Substitution of the free phenol group of compound **120** may remove bioavailability disadvantage of phenol compound such as PI-103, as demonstrated via replacement of the phenol group of PI-103 [110] with different hydrogen bond donor/acceptor heterocycles to produce bioavailable clinical drug candidates, namely VS-5584/SB2343. In view of this, Chen et al. [111] reported the synthesis and biological evaluation of a series of 6-phenylpurine-based hydroxamates as anticancer agents. Among the synthesized compounds, **123** appears to be a promising HDAC inhibitor and modulates HDAC targets in PC-3, MCF7, and MV4-11 cells in a dose-dependent manner. Despite the presence of the core structure of a potent PI3K/mTOR inhibitor (**120**), in compound **123**, it displays weak to moderate PI3K/mTOR inhibitor activity due to the blockage of the phenol group, which is crucial for PI3K/mTOR binding. Compound **123** demonstrated a broad spectrum of anti-proliferative activities against 38 tumor cell lines. In the PC-3 xenograft mode, **123** also modulated HDACs in PC-3 tumors. Besides compounds **124** and **125** are also good candidates to be further evaluated, optimized, and validated as potential anticancer agents in the series (see Figure 35).

Idelalisib has demonstrated high response rates and potency in patients with hematologic malignancies who were difficult to treat with conventional anticancer drugs and has demonstrated a tolerable safety profile in clinical trials [112]. Meanwhile, prescription information in the United States contains a black box warning for unexpected adverse effects, including diarrhea or severe and fatal colitis, hepatotoxicity, pneumonitis and intestinal perforation. To contribute to knowledge and to overcome the adverse effects of Idelalisib, Kim and colleagues [112] designed and synthesized potent phosphatidylinositol 3-kinases (PI3Kδ) inhibitors by using a purinyl functional group as the hinge binder and the phenyl group that influences the effectiveness and selectivity across the PI3K class I family as the key pharmacophores.

Compound **127** and **128** with IC_50_ values of 0.39 and 0.09 nM, respectively, exhibited excellent enzyme activity. Compound **128** was observed to be fourfold superior selectivity for PI3Kγ/δ compared to Idelalisib (**126**). Furthermore, in vivo PK experiments with **127** and **128** revealed that **128** (AUClast = 81.04 h*ng/mL, Cmax = 18.34 ng/mL, Tmax = 0.5 h, t1/2 = 10.2 h in 1 mpk dose) had improved PK. Further analyses showed that compound **127** display antitumor effectiveness in xenograft models, where PI3K pathways were properly inhibited which was confirmed by the lessening of p-AKT, p-S6, and p4EBP1 in tumor tissues. Hence, compound **127** identified as a preclinical candidate, and for further investigations.

**Figure 36:**
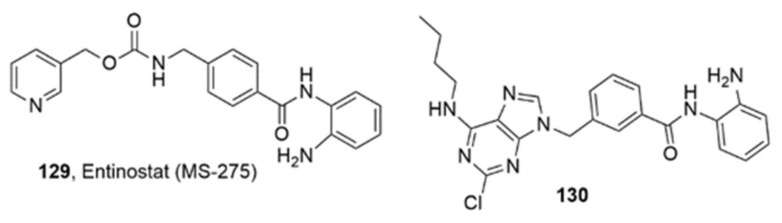
Chemical structures of new purine derivative acting as class I histone deacetylases inhibitor.

Mao et al. [113] established novel series of 9-substituted purine aminobenzamides derivatives as class I HDACs. Compound **130** exhibited excellent activity in the HDAC isoform selectivity assay. Meanwhile the potent compound is 12-fold more superior to the positive control MS-275 (**129**) against the HDAC1 isoform. MS-275 is currently in clinical trial phase II for the treatment of different types of cancers. Compound **130** showed potent antiproliferative activities on the HCT116, MDA-MB231, K562 cell lines with IC_50_ values of 0.50, 0.38, 0.12 μM, respectively. The results of SARs suggested that the optimal substituent at the C6-position of purine was butylamine group, and the short spacer was favored for HDAC inhibitory activity as well as antiproliferative effects. Compound **130** induced early apoptosis of HCT-116 cells through the intrinsic apoptosis pathway by upregulating BAX and downregulating Bcl-2, causing a notable G1/S cycle arrest in HCT-116 cells by reducing Cyclin D1, CDK2 and increasing p21 (see Figure 36).

## 4. Conclusions

This review has highlighted a number of recently published important pioneering studies involving the synthesis and biological assessment of nitrogen heterocycle compounds. Such classes of compounds as triazoles, tetrazoles, imidazoles/benzimidazoles, pyrimidines, and quinolines have been discussed in the present paper. We have only covered those studies which were published in the period of time between April 2020 and February 2022. The data presented in this review show that cyclic compounds containing the nitrogen motif are extensively represented in the literature. Even though we are reporting only a small fraction of nitrogen heterocycle compounds, most of these compounds demonstrated favorable biological and pharmacological properties, such as antibiotic, antibacterial, anti-inflammatory, antiparasitic and antitumor activities among others. This review also shows the ability of nitrogen heterocycle hybrids as a model for developing new bioactive molecules. Developing prodrug approaches to synthesize these compounds more effectively and offering options for treating various diseases sets a more exciting future for heterocyclic nitrogen hybrids. Thus, the most effective and successful application of these compounds with many future prospects is their usefulness in medicinal chemistry. Hopefully, this review will be of substantial benefit to researchers in both synthetic and medicinal chemistry fields.

## Data Availability

Not applicable.
